# Cellulose synthase* TaCESA7* negatively regulates wheat resistance to stripe rust by reducing cell wall lignification

**DOI:** 10.1007/s44154-025-00244-7

**Published:** 2025-06-16

**Authors:** Yanqin Zhang, Longhui Yu, Shuangyuan Guo, Xueling Huang, Yihan Chen, Pengfei Gan, Yi lin, Xiaojie Wang, Zhensheng Kang, Xinmei Zhang

**Affiliations:** 1 State Key Laboratory for Crop Stress Resistance and High-Efficiency Production, College of Plant Protection, Northwest A&F University, Yangling, 712100 Shaanxi China; 2 State Key Laboratory for Crop Stress Resistance and High-Efficiency Production, College of Life Sciences, Northwest A&F University, Yangling, 712100 Shaanxi China

**Keywords:** Wheat, *Puccinia striiformis* f. sp. *Tritici*, *TaCESA7*, Cellulose, Lignin, Defense responses

## Abstract

Cellulose is synthesized by cellulose synthases (CESAs) in plasma membrane-localized complexes, which act as a central component of the cell wall and influence plant growth and defense responses. *Puccinia striiformis* f. sp. *tritici* (*Pst*) is an airborne fungus that causes stripe rust to seriously endanger wheat production. In this study, a *CESA* gene, *TaCESA7*, was identified to be significantly up-regulated during *Pst* infection in wheat (*Triticum aestivum* L.). TaCESA7 was localized on the plasma membrane in dimeric form, and the dimers interact to assemble into CESA complexes. Stable overexpression of *TaCESA7* weakened the resistance of wheat to *Pst*. Knockdown of *TaCESA7* by RNA interference (RNAi) and virus-induced gene silencing led to restricted hyphal spread, increased necrotic area, and simultaneously promotes reactive oxygen species (ROS) accumulation and the expression of pathogenesis-related (PR) genes. Transcriptome analysis of *TaCESA7*-RNAi plants revealed that the up-regulated genes were significantly enriched in the phenylpropanoid biosynthesis and plant-pathogen interaction pathways. Moreover, silencing *TaCESA7* promoted the deposition of lignin and the expression of genes related to lignin synthesis. CRISPR-Cas9-mediated inactivation of *TaCESA7* in wheat could confer broad-spectrum resistance against *Pst* without affecting agronomic traits. These findings provide valuable candidate gene resources and guidance for molecular breeding to improve the resistance of wheat to fungal disease.

**Supplementary Information:**

The online version contains supplementary material available at 10.1007/s44154-025-00244-7.

## Introduction

In response to pathogens, plants have evolved a plethora of resistance mechanisms, which are either constitutively expressed or induced after pathogen attack (Couto et al. 2016; Ding et al. 2022). One common resistance mechanism to all plant cells is the presence of a cell wall (Wolf et al. 2012). The cell wall acts as the first physical barrier to defend against pathogen invasion, which also plays a role in sensing external stresses and transferring the corresponding signal to stimulate defense responses (Cosgrove 2005, 2024). According to different developmental stages, the cell wall can be divided into the primary cell wall (PCW) formed in early cell development and the secondary cell wall (SCW) formed inside the PCW after cell morphogenesis (Cosgrove 2005). The PCW is mainly composed of cellulose, pectin, hemicellulose, and some methylated, esterified, and acetylated forms of these compounds, whereas the SCW is mainly composed of cellulose that is rich in lignin and xylan (Kim et al. 2020; Zhang et al. 2021). Modifications of cell wall composition and structure take place during plant development but also as a consequence of exposure to pathogen attack. These modifications impact cell wall integrity (CWI) and can initiate molecular adaptive mechanisms, such as cell wall composition remodeling and activation of defensive responses (Bacete et al. 2018; Vaahtera et al. 2019). The dynamic remodeling of these cell wall components plays a crucial role in regulating plant resistance (Xia et al. 2024).

Cellulose is the main load-bearing component of both PCW and SCW conferring strength to plant cells (Höfte et al., 2017). It is synthesized at the plasma membrane by the catalytic subunits of the cellulose synthase (CESA) protein complex (CSC), which plays a central role in determining the mechanical properties of plant cell walls (Persson et al. 2007). The CSC is composed of 18–24 subunits of CESA, which work together to catalyze the formation of glycosidic bonds to produce a cellulose microfibril (Purushotham et al. 2020; Turner and Kumar 2018). CESA proteins are highly conserved among plant species, sharing common domains and motifs, including zinc fingers and eight transmembrane domains (Kumar et al. 2016a, b; Pear et al. 1996). In *Arabidopsis thaliana*, 10 CESA genes have been identified. AtCESA1, AtCESA3, and AtCESA6 synthesize PCW cellulose, while AtCESA4, AtCESA7, and AtCESA8 comprise the CSCs necessary for SCW cellulose production (Taylor 2008). The remaining genes, AtCESA2, 5 and 9, are partially redundant with AtCESA6 (Persson et al. 2007; Desprez et al. 2007). AtCESA10 remains uncharacterized. Maize, rice, and barley possess 13, 11 and 9 CESA genes, respectively (Zhang et al. 2014; Houston et al. 2015; Wang et al. 2010). Mapping studies from Arabidopsis, maize and rice revealed that the members of the *CESA* gene family were spread across the genome although some genes were clustered together (Holland et al. 2000). Bioinformatics and mutational analyses have shown that OsCESA1, 3, and 8 and OsCESA4, 7, and 9 are involved in the cellulose synthesis of the PCW and SCW in rice, respectively (Li et al. 2017; Wang et al. 2010). However, the functions of the *CESA* gene family in wheat have still been rarely reported.

Mutations in *CESAs*, whether affecting PCW or SCW formation, elicit various stress and defense responses, bolstering resistance against specific pathogens (Hématy et al. 2009; Doblin et al. 2002). For example, a *CESA3*-deficient mutant of Arabidopsis shows increased resistance against infection by the pathogens *Pseudomonas syringae*, *Botrytis cinerea*, and *Erysiphe cichoracearum* (Ellis et al. 2001; Ellis et al. 2002). The Arabidopsis mutant *irx5/3/1* (irregular xylem) with disruption of *CESA4/7/8* displays structural changes in the SCW that are accompanied by enhanced disease resistance against the bacterial pathogen *Ralstonia solanacearum* and the necrotrophic fungal pathogen *Plectosphaerella cucumerina* (Hernández-Blanco et al. 2007). The expression of *CESA4/7/8* is positively regulated by the transcription factor *MYB46*. Consistent with the role of *CESA4/7/8* in plant disease resistance, a loss-of-function mutation of *MYB46* increased the disease resistance of Arabidopsis to *B. cinerea* (Ramírez et al. 2011). However, blocking the cellulose synthesis pathway decreases plant disease resistance. For instance, when *HvCSLD2*, encoding cellulose synthase-like D, was silenced, barley had a reduced cellulose content in the epidermal cell wall and its papillae were consequently more easily penetrated by the fungus *Blumeria graminis*, leading to compromised resistance to powdery mildew (Douchkov et al. 2016). Therefore, altering expression or mutating the *CESA* genes has a specific impact on CWI and results in the release of damage-associated molecular patterns (DAMPs) and activation of immune signaling, leading to either pathogen susceptibility or resistance (Bacete et al. 2018).

The reduction in cellulose synthesis triggers the activation of the CWI pathway. This induces the expression of lignin synthesis genes, leading to enhanced cell wall lignification (Zhong et al. 2011). Lignin is another major component of the SCW that forms the main physical barrier in the defense response to pathogen infection (Zhao et al. 2014; Cesarino 2019; Ma 2024). The monolignol subunits of lignin are derived from phenylalanine through several enzymatic reactions in the core phenylpropanoid pathway involving a series of enzymes (Coomey et al. 2020; Gomez-Cano et al. 2020). Overexpression of the rice 4 CL gene *OsAAE3* decreased lignin accumulation and increased the sensitivity to rice blast, which may be related to the decrease of peroxidase activity and expression of pathogen-related 1a (*PR1*) (Liu et al. 2017). Inhibition of phenylalanine ammonia-lyase (PAL) can not only reduce the lignin content but also change the content of phenylpropane and its derivatives such as salicylic acid, which can cooperatively change the disease resistance of plants. In the Arabidopsis* ref8* (reduced epidermal fluorescence 8) mutant, the activity of the enzyme coumaroyl shikimate 3-hydroxylase was reduced, accompanied by reduction in the plant’s resistance to fungal infection (Franke et al. 2002). Moreover, knockout of the lignin pathway gene *BnF5H* decreased the S/G lignin compositional ratio and improved *Sclerotinia sclerotiorum* resistance in *Brassica napus* (Cao et al. 2021). Brown midrib maize harbors mutations that result in lower lignin levels, making the plant more susceptible to foliar fungal, anthracnose leaf blight, and bacterial (Stewart’s wilt) diseases (Kolkman et al. 2023).

Stripe rust is caused by *Puccinia striiformis* f. sp. *Tritici* (*Pst*), which is one of the most destructive fungal diseases in wheat (Hovmøller et al. 2010). However, the contribution of the *CESA* gene family during the interaction between wheat and stripe rust has not been established. In this study, we reported that a cellulose synthase *TaCESA7* localized on the plasma membrane was significantly induced in the affinity interaction of wheat with the fungal pathogen *Pst* causing stripe rust. Functional characterization revealed that *TaCESA7* acts as a susceptibility factor in wheat-*Pst* interactions, and transcriptome analysis also demonstrated that knockdown of *TaCESA7* enhanced the expression of defense-related genes and phenylpropanoid biosynthesis genes in *TaCESA7*-RNAi plant. Further research showed that the content of cellulose decreased, while the content of lignin increased significantly, and the degree of lignification deepened in *TaCESA7*-RNAi plants, suggesting that the remodeling of cell wall components in *TaCESA7* silencing materials improved resistance of wheat. Moreover, knockout of *TaCESA7* in wheat could confer broad-spectrum resistance against *Pst* without affecting agronomic traits. These findings provide valuable candidate gene resources and guidance for molecular breeding to improve the resistance of wheat to fungal disease.

## Results

### TaCESA7 is upregulated by Pst infection and forms plasma membrane-localized homodimers and heterodimers with TaCESA8

**Fig. 1:**
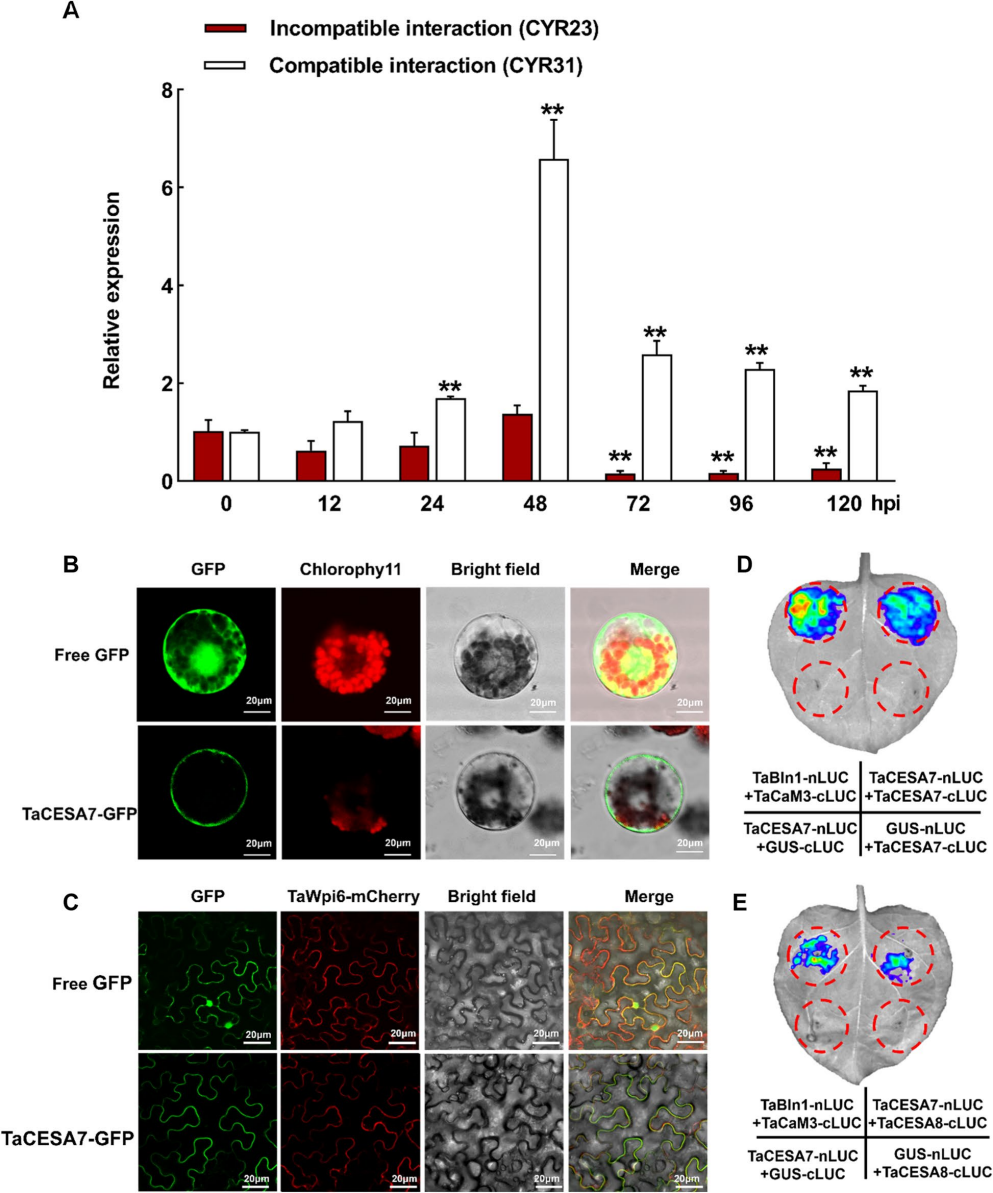
The expression level of *TaCESA7* in response to the infection of *Pst* and the formation of dimers with TaCESA8 in the plasma membrane. **A** *Pst* infection significantly induced the expression of *TaCESA7*. The leaves of wheat Suwon 11 were inoculated with CYR23 and CYR31 at 0, 12, 24, 48, 72, 96, and 120 hpi. Untreated leaves served as control. **B** Localization analysis of TaCESA7 in wheat protoplasts. Free GFP as a control. GFP fluorescence is green. Red fluorescence includes chlorophyll auto-fluorescence. All signals are monitored using confocal microscope FV3000. Scale bars = 20 μm. **C** Localization analysis of TaCESA7 in *N. benthamiana* leaves. TaWpi6-mCherry as a marker of the plasma membrane. All signals are monitored using confocal microscope FV3000. Scale bars = 20 μm. **D** Split-luciferase assays determined the interaction of TaCESA7 and TaCESA7 in *N. benthamiana* leaves. **E** Split-luciferase assays determined the interaction of TaCESA7 and TaCESA8 in *N. benthamiana* leaves. TaBln1-nLUC and TaCaM3-cLUC was used as positive control. GUS-nLUC and GUS-cLUC was used as negative control. The relative expression of *TaCESA7* was calculated using the comparative threshold method (2^−ΔΔCT^). Expression levels were normalized to *TaEF*. Values in this figure represent the mean ± SD of three independent replicates. Asterisks indicate that the difference in relative expression of *TaCESA7* at that time point was significant (**P < 0.01) by two-tailed Student’s *t-*test

CESAs are essential enzymes for the formation of plant cell walls and play an important role in the plant defense response against pathogens (Arya et al. 2021). In this study, we documented 24 *CESA* genes from wheat in the Ensembl Plants database with three paralogs in the homoeologous genomes A, B, and D, named *TaCESA1-8*. The Ensembl IDs of all identified wheat *CESA* genes are given in Table S1. To confirm whether *TaCESA*s are involved in the response of wheat to *Pst*, we used reverse transcription-quantitative polymerase chain reaction (RT-qPCR) to analyze the transcription levels of the *TaCESA*s in wheat inoculated with the avirulent *Pst* race CYR23 (incompatible interaction) and the virulent race CYR31 (compatible interaction) at different time points (Fig. S1). The up-regulated expression of *TaCESA7* was most significant after CYR31 infection by using conservative primers (Fig. 1A). Further analysis of the expression levels of *TaCESA7* homologs from subgenomes A, B, or D revealed that although the expression levels were different, the change trend at each time is basically the same (Fig. S2). These findings suggested that *TaCESA7* may play a certain role in the wheat-*Pst* interaction.

BLAST analysis identified three TaCESA7 copies (3 A/3B/3D) with 98.83% nucleotide and 99.53% amino acid similarity, implying functional redundancy (Fig. S3 A). TaCESA7 encodes a 983-amino acids protein (2952-bp CDS, coding sequence; Fig. S3B) featuring conserved CESA family domains, including eight transmembrane regions and an N-terminal zinc finger-like motif (Fig. S3 C, D). Phylogenetically, TaCESA7 clusters with OsCESA4 and AtCESA8, known cellulose synthase subunits (Fig. S4 and Table S1) Subcellular localization via TaCESA7-GFP in wheat protoplasts and tobacco leaves confirmed plasma membrane targeting, co-localizing with the membrane marker TaWpi6-mCherry (Fig. 1B, C). Studies have shown that CESA4, 7, and 8 are required for the formation of functional CSCs during secondary wall cellulose synthesis (Taylor et al. 2004). Subcellular localization indicates that TaCESA4 and TaCESA8 are located in the plasma membrane and cytoplasm (Fig. S5 A). In order to analyze the relationship between the secondary cell wall synthesis component TaCESA4/7/8 in wheat, their interaction was confirmed in split-luciferase assays. The results showed that TaCESA7 self-interaction and heterodimerization with TaCESA8, but not TaCESA4 (Fig. 1D, E and Fig. S5B). These findings suggest TaCESA7, like OsCESA4 and AtCESA8, contributes to cellulose synthesis via plasma membrane-localized homodimers and TaCESA8 heterodimers, forming functional CSCs.

### Overexpression of TaCESA7 enhances the susceptibility of wheat to Pst

**Fig. 2:**
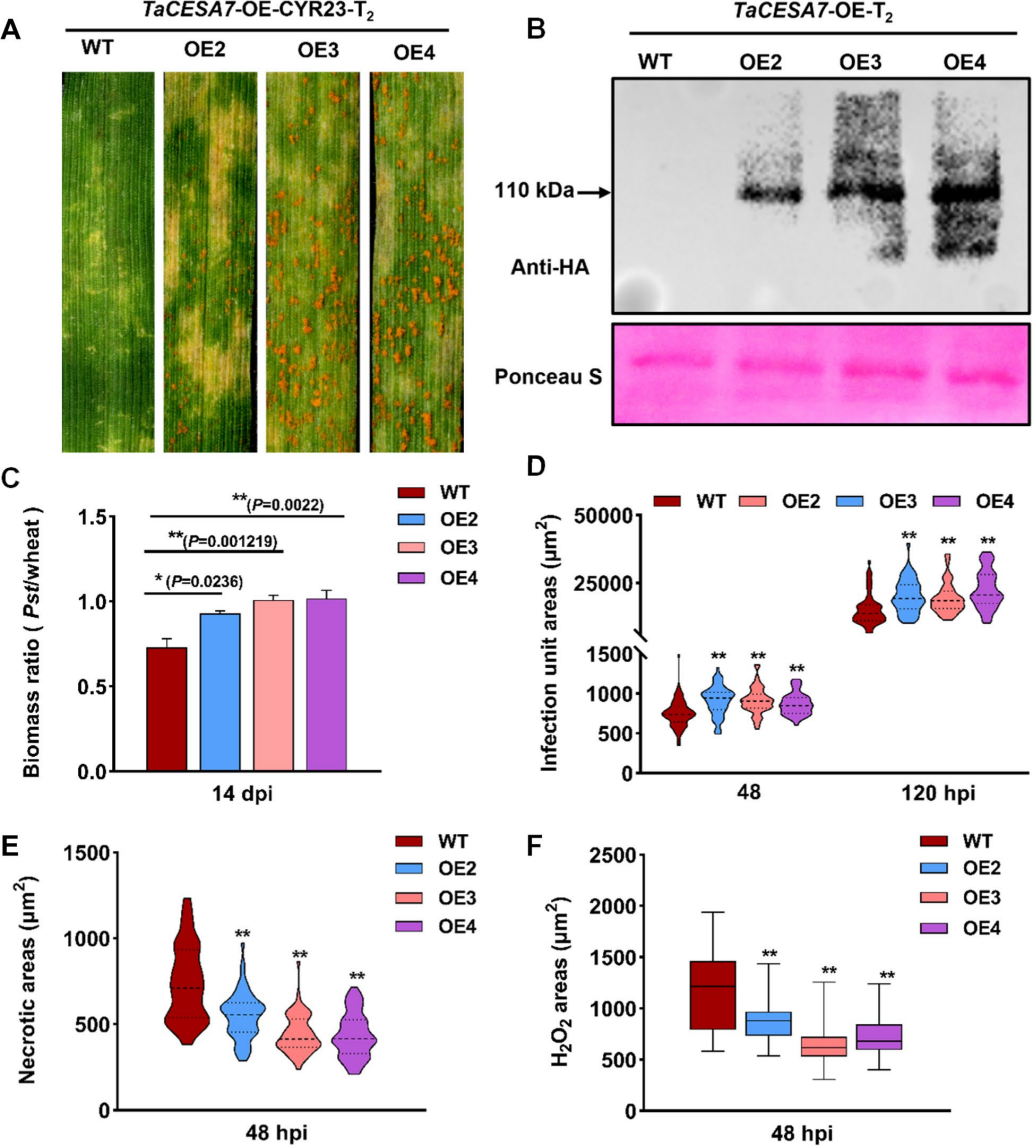
Overexpression of *TaCESA7* promotes wheat susceptibility to *Pst*. **A** Disease phenotypes of the second leaves of *TaCESA7*-OE wheat plants and WT plants infected at 14 dpi with *Pst*. **B** Protein expression of TaCESA7-HA recombinant protein in *TaCESA7*-OE transgenic plants; Ponceau S, Ponceau stain of Rubisco. **C** Ratio of fungal to wheat nuclear content determined using the contents of fungal *PsEF* and wheat *TaEF* at 14 dpi. **D** Infection area per infection site in WT and *TaCESA7* overexpression plants infected with CYR23 at 48 and 120 hpi. Fifty infected sites were measured in each replicate. **E** Quantification of necrotic cell death area in different *TaCESA7*-OE transgenic lines at 48 hpi. Fifty infected sites were measured in each replicate. **F** Quantification of H_2_O_2_ accumulation area in different *TaCESA7*-OE transgenic lines at 48 hpi. Values in this figure represent the mean ± SD of three independent biological replicates (50 infection sites each time). Asterisks indicate significant differences relative to the WT plants by two-tailed Student’s *t-*test (*P < 0.05, **P < 0.01)

To confirm the function of *TaCESA7* in the wheat defense response against *Pst*, we developed transgenic lines in the wild type (WT) background in which *TaCESA7* was overexpressed. Three T_2_ transgenic overexpression (OE) lines (OE2, OE3 and OE4) were successfully generated and confirmed by PCR amplification and RT-qPCR (Fig. S6). When the *TaCESA7*-OE lines and WT were inoculated with the avirulent *Pst* isolate CYR23. respectively, *TaCESA7*-OE lines displayed substantial sporulation and necrotic spots indicative of the typical hypersensitive response (HR) phenotype, and only sporadic pustules were detected at 14 days post-infection (dpi) of *Pst* (Fig. 2A). and western blotting analysis confirmed that the expression of *TaCESA7* was overexpressed (Fig. 2B). The fungal biomass was also increased compared with that in the WT plants at 14 dpi (Fig. 2C). In addition, the infection areas, the length of the hyphae, the numbers of hyphal branches, and haustorial mother cells were significantly increased in the *TaCESA7*-OE plants (Fig. 2D and Fig. S7 A-D). Reactive oxygen species (ROS) accumulation (Fig. 2E) and cell death (Fig. 2F) at the infection sites of *TaCESA7*-OE plants triggered by CYR23 were significantly suppressed at 48 h post-infection (hpi) (Fig. S7 A). The transcription of the pathogenesis-related genes *TaPR1* and *TaPR2* was significantly suppressed in the *TaCESA7*-OE plants at 24 and 48 hpi (Fig. S7E, F). These results indicated that *TaCESA7* negatively regulates wheat resistance to *Pst*.

### Silencing TaCESA7 reduces the susceptibility of wheat to Pst

**Fig. 3:**
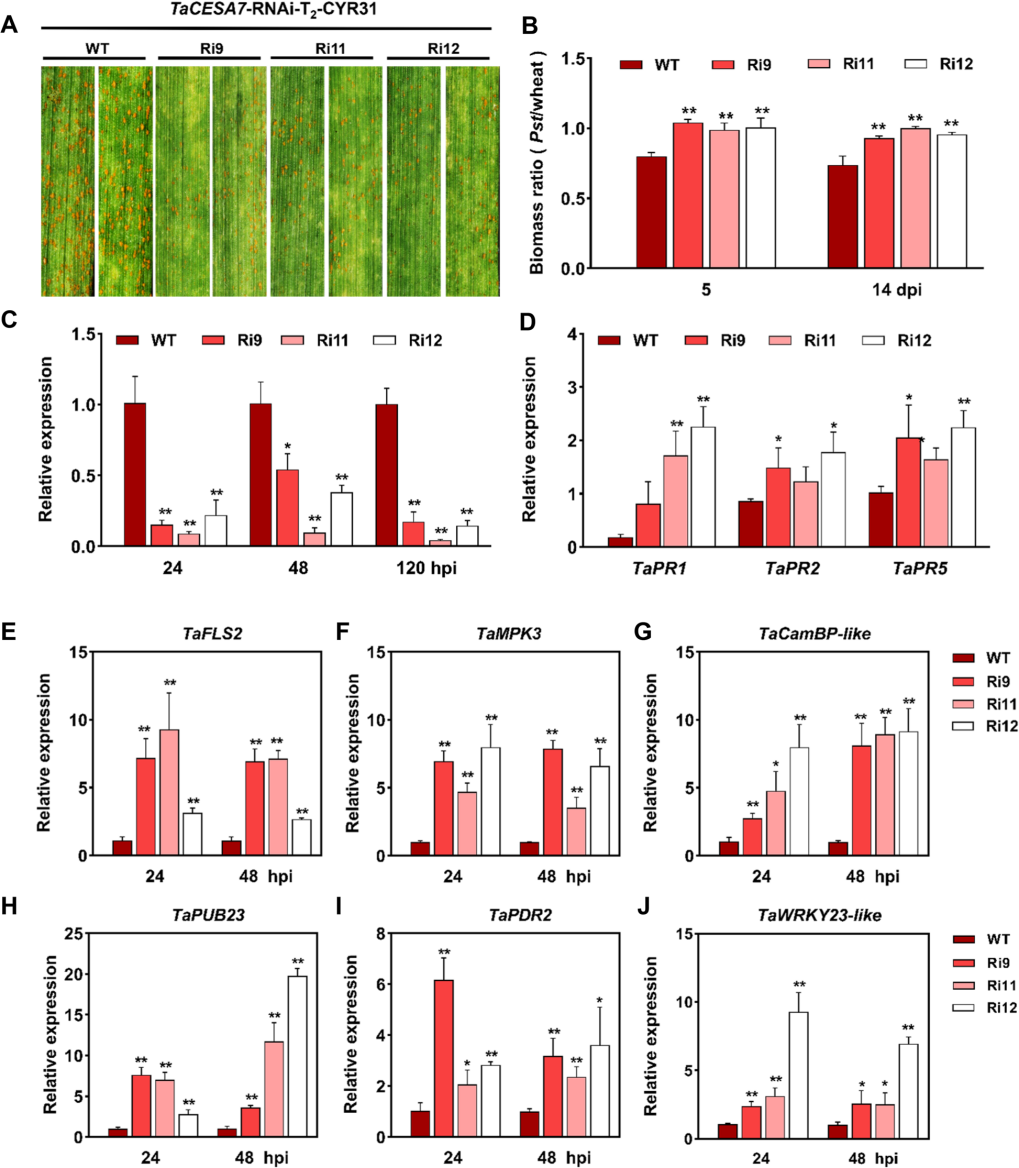
*TaCESA7*-RNAi plants enhanced wheat resistance to *Pst*. **A** Stripe rust disease symptoms on *TaCESA7*-RNAi and WT leaves inoculated with the virulent *Pst* race CYR31 at 14 dpi. **B** The biomass ratio of total DNA in CYR31-infected wheat leaves (*Pst*/wheat) at 5 and 14 dpi was determined using RT-qPCR. The data were normalized to *TaEF* and *PsEF*. **C** Silencing efficiency of *TaCESA7* in *TaCESA7*-RNAi transgenic wheat lines and WT plants at 24, 48 and 120 hpi. The expression of *TaCESA7* in WT plants was set to “1”. **D** The expression profiles of pathogenesis-related genes *TaPR1*, *TaPR2* and *TaPR5* in *TaCESA7*-RNAi transgenic wheat lines and WT plants at 48 hpi. **E-J** Transcript levels of PTI marker genes in *TaCESA7*-RNAi plants at 24 and 48 hpi. *TaFLS2* (**E**), *TaMPK3* (**F**), *TaCamBP-like* (**G**), *TaPUB23* (**H**), *TaPDR2* (**I**), and *TaWRYK23-like* (**J**) were measured by RT-qPCR. Values in this figure are the mean ± SD of three independent replicates. Asterisks indicate significant differences (*P < 0.05; **P < 0.01) relative to the WT plants by two-tailed Student’s *t-*test

To further investigate the function of *TaCESA7* in susceptibility to stripe rust, we employed both transient virus-induced gene silencing (VIGS) and stable RNAi approaches. BSMV:*TaCESA7*-as plants (1as/2as constructs; Fig. S8 A) exhibited reduced *Pst* spore counts, fungal mRNA levels and biomass (Fig. S8B-E). Histological analysis of the *TaCESA7-*silenced plants showed a significant increase in the size of colony area at the infection site (Fig. S8 F-H). Three independent *TaCESA7-*RNAi lines (Ri9/Ri11/Ri12; Fig. S9 A) exhibited significantly lower *Pst* spore counts (Fig. 3A) and fungal biomass (Fig. 3B) compared to WT plants. which was consistent with the results from VIGS. RT-qPCR confirmed that the transcription levels of *TaCESA7* were significantly inhibited at 24, 48, and 120 hpi (Fig. 3C). Meanwhile, compared with that of WT plants, the relative expression of disease-related genes *TaPR1*, *TaPR2,* and *TaPR5* in *TaCESA7*-silenced plants was significantly up-regulated at 24 dpi (Fig. 3D). In addition, transcript levels of the pattern-triggered immune responses (PTI) marker genes *TaFLS2*, *TaMPK3*, *TaCamBP-like*, *TaPUB23*, *TaPDR2*, and *TaWRYK23-like* significantly increased in *TaCESA7*-RNAi plants at 24 and 48 hpi (Fig. 3E-J).

**Fig. 4:**
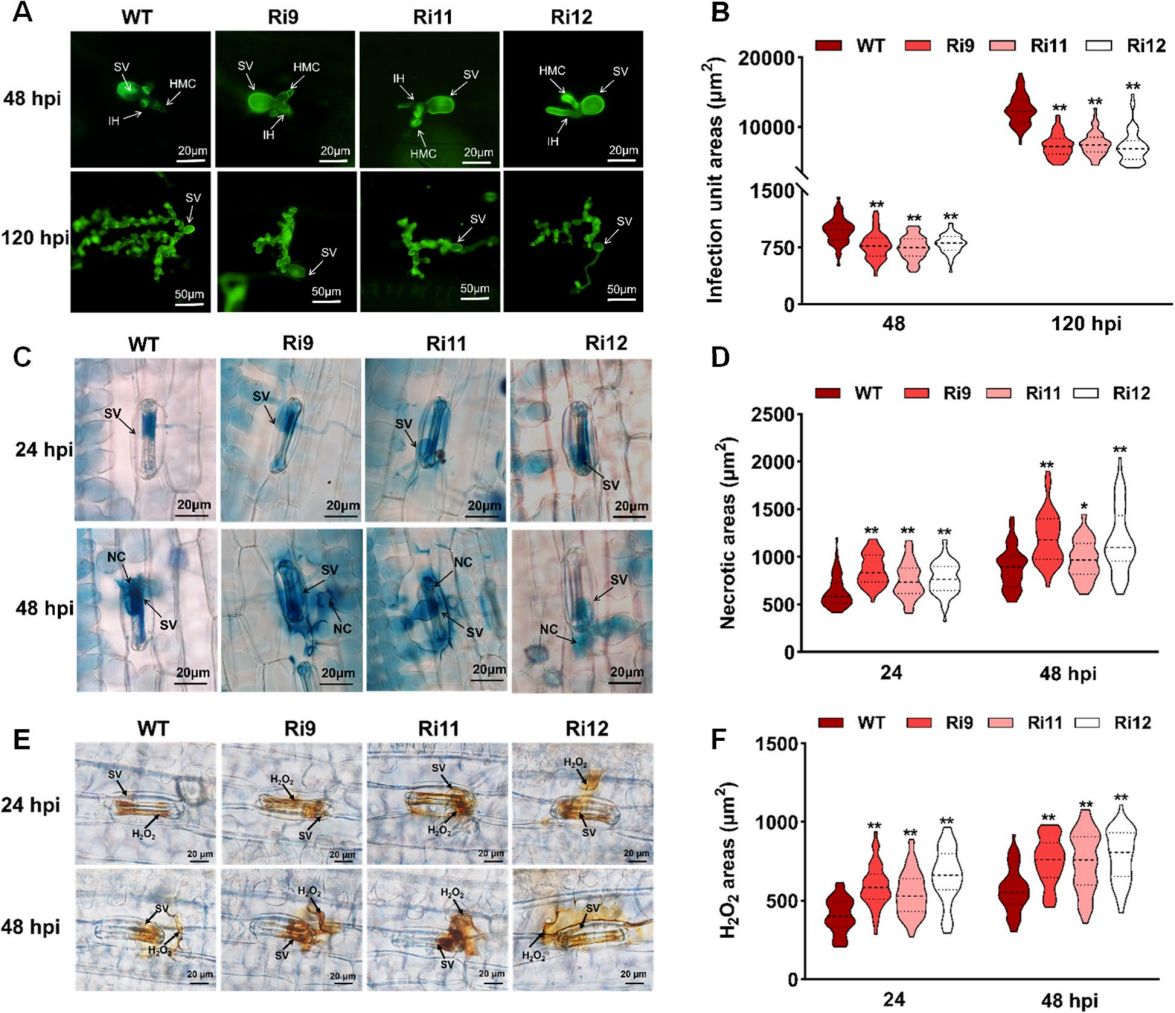
Histological observation of fungal growth and host defense in *TaCESA7*-RNAi plants. **A** Mycelial development of *TaCESA7*-RNAi and WT plants at 48 and 120 hpi. *Pst* infected structures were stained with WGA (Wheat germ agglutinin) and visualized under a fluorescence microscope. SV, substomatal vesicles; IH, infected hyphae; HMC, haustoria mother cells. 48 hpi, Bar = 20 µm; 120 hpi, Bar = 50 µm. **B** Infected area of *Pst* at 48 and 120 hpi in *TaCESA7*-RNAi plants was measured using CellSens Entry software. Fifty infected sites were measured in each replicate. **C** Representative pictures of necrotic cell death in *Pst* infected wheat cells detected by trypan blue staining in *TaCESA7*-RNAi lines at 24 and 48 hpi. SV, substomatal vesicle; NC, necrotic cell. Bar = 20 µm. **D** Quantification of necrotic cell death area in different transgenic lines at 24 and 48 hpi. Fifty infected sites were measured in each replicate. **E** Representative pictures of H_2_O_2_ accumulation by DAB staining in wheat leaves at 24 and 48 hpi after infection with CYR31. Samples were observed under a fluorescence microscope. SV, substomatal vesicle. Bar = 20 µm. **F** The area of H_2_O_2_ accumulation at each site of infection was measured at 24 and 48 hpi. Fifty infected sites were measured in each replicate. Values in this figure represent the mean ± SD of three independent samples. Asterisks indicate significant differences compared with WT plants (*P < 0.05; **P < 0.01) using the two-tailed Student's *t-*test

To clarify the enhanced disease resistance phenotypes of the *TaCESA7*-RNAi wheat seedlings, the fungal development and host response were further evaluated using fluorescence microscopy (Fig. 4A). The *Pst* infection areas of the *TaCESA7*-RNAi plants were significantly reduced at 48 and 120 hpi compared with those in control plants (Fig. 4B). The necrotic area of *TaCESA7*-RNAi plants significantly increased in 24 and 48 hpi of infection with *Pst* by trypan blue staining (Fig. 4C, D). We further analyzed the extent of ROS accumulation in *TaCESA7*-RNAi plants after inoculation with *Pst* CYR31. 3,3′-diaminobezidine (DAB) (Fig. 4E, F) showed that H_2_O_2_ accumulation were significantly increased at the infection sites the *TaCESA7*-RNAi plants compared with those in WT plants. Previous studies have shown that plant mutants impaired in cellulose synthesis exhibit severe developmental defects such as dwarfing and reduced yield (Schulze et al. 2010). Therefore, we assessed the effect of *TaCESA7* knockdown on the key agronomic traits of wheat including plant height, ear length, tiller number, thousand-grain weight, grain width, and grain length in the field. The wheat growth, yield, and other agronomic traits were similar between *TaCESA7*-RNAi and WT (Fig. S9B-I), indicating that knockdown of *TaCESA7* does not have an obvious impact on the major agronomic traits of wheat. Collectively, these results of *TaCESA7* suppression indicated that it substantially contributes to the severity of *Pst* infection of wheat.

### Global gene expression analysis reveals changes in the expression of defense-related genes and phenylpropanoid biosynthesis genes

**Fig. 5:**
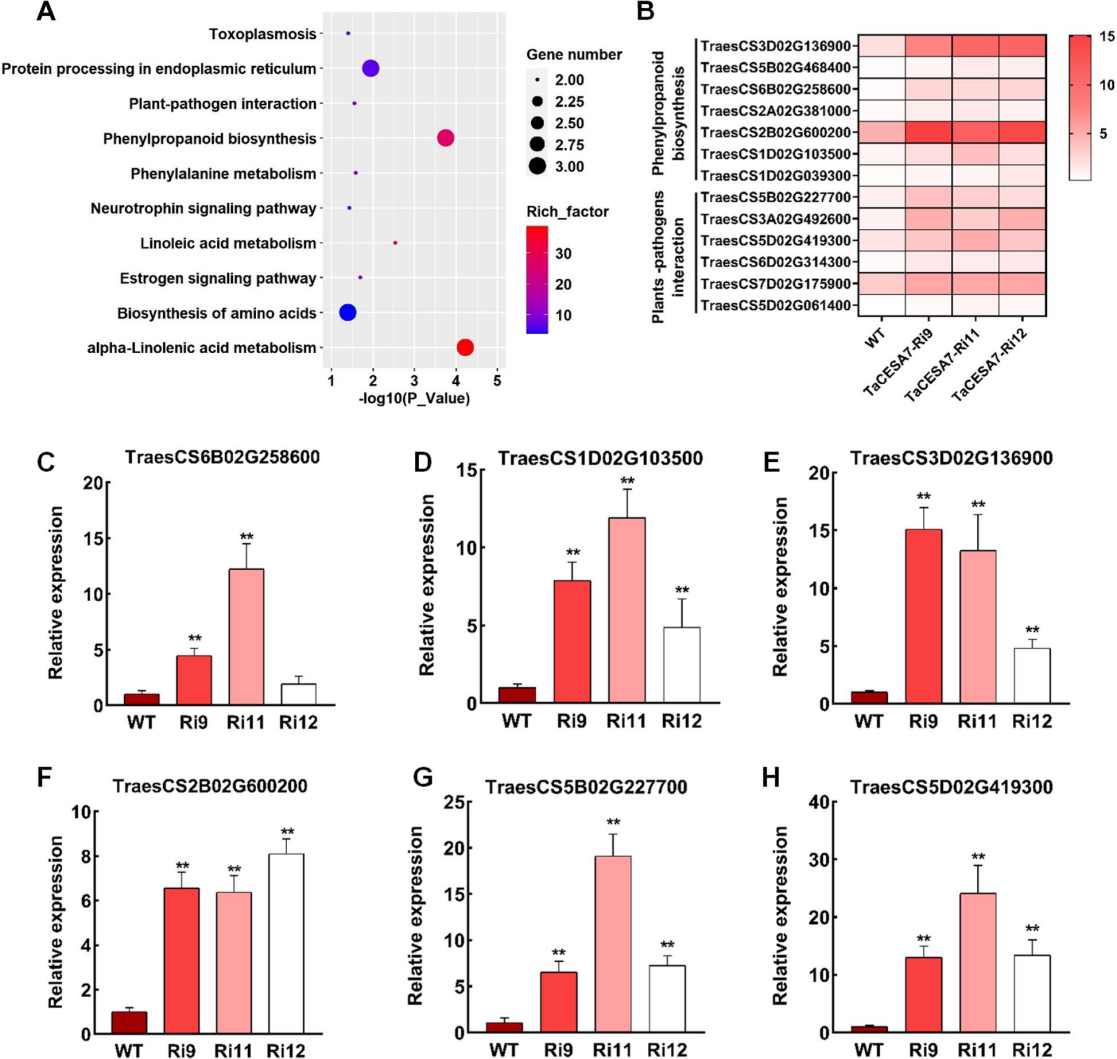
Knockdown of *TaCESA7* causes global transcriptional reprogramming in transgenic wheat. **A** The number of differentially expressed genes (DEGs) contained in each KEGG pathway in the *TaCESA7*-RNAi transgenic lines based on up-regulated genes. **B** Heat map analysis of the expression of up-regulated DEGs enriched in the pathways of phenylpropanoid biosynthesis and plant-pathogen interaction in the *TaCESA7*-RNAi transcriptome. **C-H** The expression levels of six up-regulated DEGs of the ‘phenylpropanoid biosynthesis’ and ‘plant-pathogen interaction’ pathways from RNA-seq data were selected for RT-qPCR. Values in this figure are the mean ± SD of three independent replicates. Asterisks indicate significant differences (**P < 0.01) relative to the WT plants using the two-tailed Student’s *t*-test

To better understand the mechanism of *TaCESA7*-mediated wheat resistance to *Pst*, RNA-sequencing (RNA-seq) analysis of the leaves of WT and *TaCESA7*-RNAi plants was performed. Transcriptome comparison identified 318 differentially expressed genes (DEGs) in *TaCESA7*-RNAi transgenic wheat compared with non-transgenic WT seedlings, including 234 up-regulated and 84 down-regulated genes in the transgenic plants (Fig. S10 A-C and Table S3). GO enrichment analysis of DEGs were mainly enriched in oxylipin metabolic process, phenylalanine ammonia-lyase activity and protein refolding (Fig. S11). Kyoto Encyclopedia of Genes and Genomes (KEGG) pathway enrichment analysis suggested that the down-regulated DEGs were mainly enriched in the cyanoamino acid metabolism, plant hormone signal transduction, arachidonic acid metabolism, and peroxisome pathways (Fig. S12), while the up-regulated genes were significantly enriched in the plant-pathogen interaction, phenylpropanoid biosynthesis, phenylalanine metabolism, alpha-linolenic acid metabolism, and linoleic acid metabolism pathways (Fig. 5A). Notably, many DEGs were enriched in the phenylpropanoid biosynthesis and plant-pathogen interaction pathways (Fig. 5B). To confirm the reliability of the RNA-seq data, six DEGs enriched in the phenylpropanoid biosynthesis and plant-pathogen interaction pathways were selected for RT-qPCR verification (Fig. 5C-H). The changes in expression levels of these six genes were consistent with the RNA-seq data, suggesting that silencing of *TaCESA7* possibly modulates wheat resistance through enhancing the expression network of defense-related genes and phenylpropanoid biosynthesis genes.

### Silencing of TaCESA7 promotes lignin accumulation to enhance wheat disease resistance

**Fig. 6:**
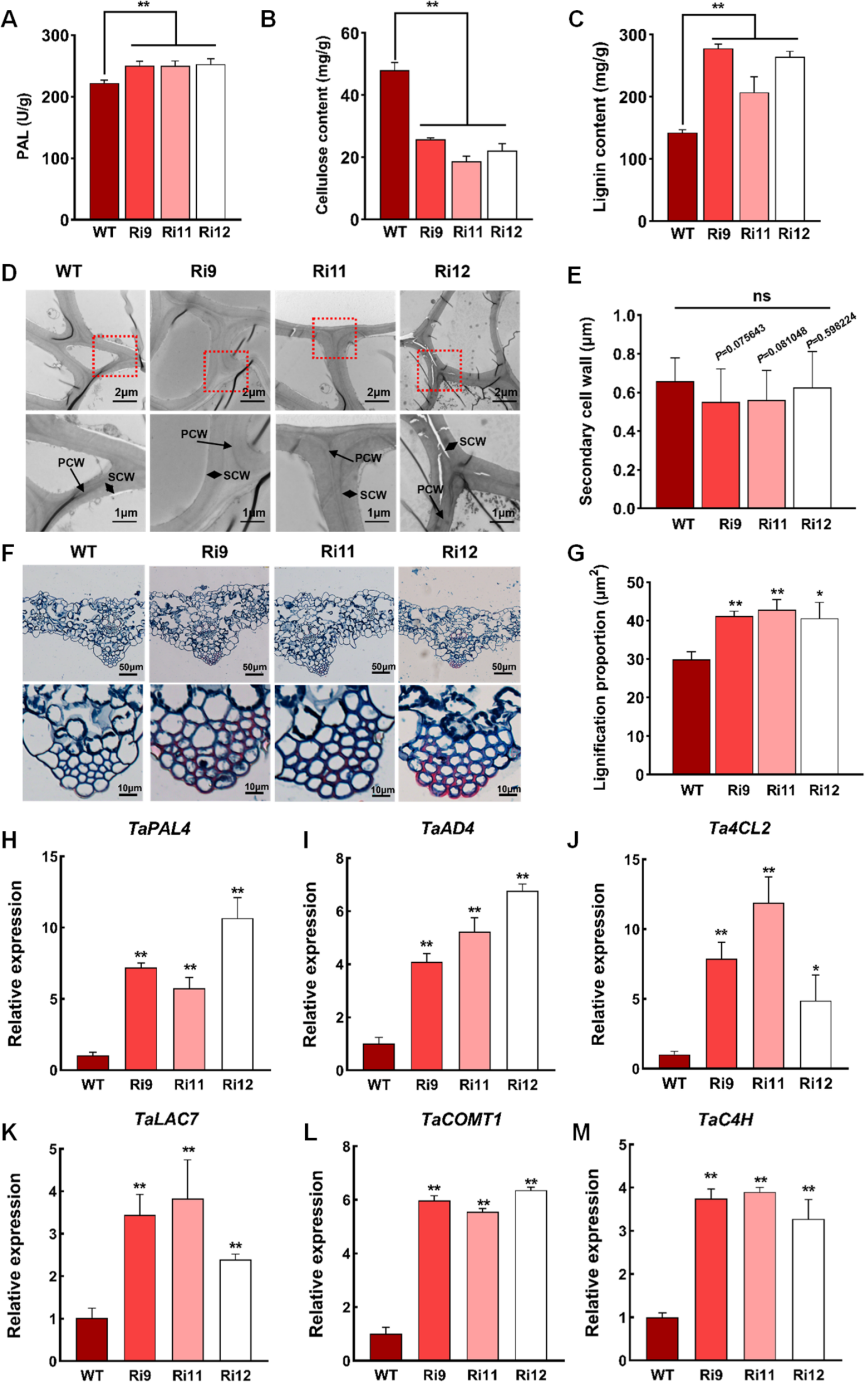
Silencing of *TaCESA7* promotes lignin accumulation to enhance wheat disease resistance. **A** PAL (phenylalanine ammonia lyase) activity was increased in *TaCESA7*-RNAi transgenic plants at 24 hpi by *Pst*. **B** The cellulose content of the *TaCESA7*-RNAi transgenic wheat leaves compared to that in the WT plants at 24 hpi. **C** The lignin content of the *TaCESA7*-RNAi transgenic wheat leaves compared to that in the WT plants at 24 hpi. **D** The cell wall of *TaCESA7*-RNAi plant infected by stripe rust at 24 hpi was observed by transmission electron microscopy (TEM). *n* = 30. Bar = 1 µm and 2 µm. PCW, primary cell wall; SCW, secondary cell wall. **E** Data are means of the secondary cell wall thickness from three independently replicated experiments as determined using the ImageJ software. Data are represented as the mean ± SD (*n* = 20). ns, no statistically significant difference (**F**) The leaves of *TaCESA7*-RNAi material were sliced in paraffin and stained with safranine and solid green at 24 hpi by stripe rust. Bar = 10 µm and 50 µm. **G** The degree of lignification of the *TaCESA7*-RNAi plant at 24 hpi. The cell wall lignification of 30 leaves was measured using ImageJ in each replication. **H-M** The expression of lignin biosynthesis genes *TaPAL4* (**H**), *TaAD4* (**I**), *Ta4 CL2* (**J**), *TaLAC7* (**K**), *TaCOMT1* (**L**), and *TaC4H* (**M**) in *TaCESA7*-RNAi transgenic plants at 24 hpi. Values in this figure are the mean ± SD of three independent replicates. Asterisks indicate significant differences (*P < 0.05, **P < 0.01) relative to the WT plants using the two-tailed Student’s *t*-test

Previous studies indicated that the phenylpropanoid biosynthesis pathway is involved in disease resistance and immunity by promoting the synthesis and accumulation of lignin (Huang et al. 2010; Yadav et al. 2020). The PAL activity of *TaCESA7*-RNAi plants increased significantly (Fig. 6A). *TaCESA7* is involved in the synthesis of the SCW, which is composed of cellulose, lignin, and hemicellulose (Kaur et al. 2016). Therefore, to elucidate the molecular mechanism by which silencing *TaCESA7* enhances disease resistance, we examined the cell wall components of *TaCESA7*-RNAi plants. The leaves of the *TaCESA7*-RNAi transgenic wheat showed a 38–53% reduction in cellulose levels (Fig. 6B) and a 43.5–60% increase in lignin levels compared with those of WT plants (Fig. 6C). To investigate the effects of changes in lignin and cellulose contents in *TaCESA7-*silenced leaves on the cell wall structure, we inoculated *TaCESA7*-RNAi leaves with *Pst* for 24 hpi. Transmission electron microscopy showed that the SCW of *TaCESA7*-RNAi plants did not change significantly compared with that of WT plants (Fig. 6D, E). Safranine solid green staining of the plant tissue (Fig. 6F) demonstrated that the degree of lignification of the *TaCESA7*-RNAi plants was significantly higher than that of the WT (Fig. 6G). Furthermore, the *TaCESA7*-RNAi transgenic plant exhibited significantly up-regulated expression of the lignin biosynthesis genes *TaPAL4*, *TaAD4*, *Ta4 CL2*, *TaLAC7*, *TaCOMT1*, and *TaC4H* after inoculation with *Pst* CYR31 (Fig. 6H-M). These results indicate that silencing *TaCESA7* induces lignin accumulation, thereby enhancing the disease resistance of wheat.

### CRISPR-Cas9 editing of TaCESA7 confers broad-spectrum stripe rust resistance

**Fig. 7:**
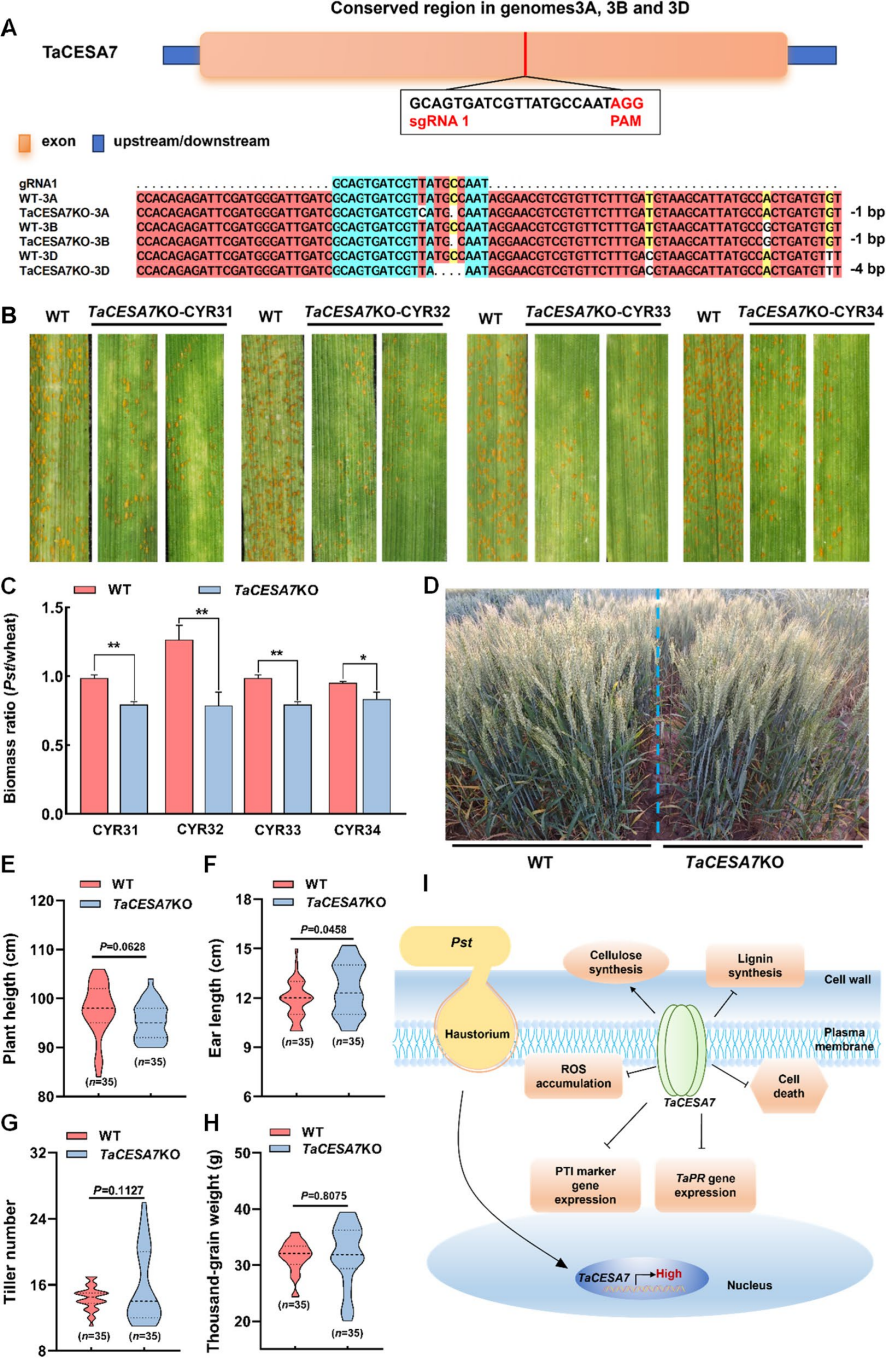
CRISPR-Cas9 editing of *TaCESA7* confers broad spectrum stripe rust resistance. **A** Schematic diagram of *TaCESA7* gene structure and the sequences of the a sgRNA designed to target the three homoeoalleles (3 A, 3B, 3D), of *TaCESA7* for editing by CRISPR-Cas9. Pink rectangle, exon; Blue rectangle, upstream/downstream. Red line, sgRNA. **B** The disease phenotypes of *TaCESA7*KO and WT (Fielder) inoculated with *Pst* races CYR31**,** CYR32, CYR33, and CYR34. **C** Detection of fungal biomass in *TaCESA7*KO plants after *Pst* infection at 14 dpi. **D** The growth phenotype of *TaCESA7 KO* and WT plants in the field. **E–H** Comparison of (**E**) plant height (*n* = 35), (**F**) ear length (*n* = 35), (**G**) tiller number (*n* = 35), and (**H**) thousand-grain weight (*n* = 35) in WT and *TaCESA7*KO plants under normal growth conditions. **I** Working model for the function of *TaCESA7* during wheat-*Pst* interaction. During wheat infection by *Pst*, *TaCESA7* expression is significantly upregulated. Silencing *TaCESA7* reduces cellulose biosynthesis, thereby activating the CWI pathway, which markedly promotes lignin biosynthesis and enhances cell wall lignification. Additionally, *TaCESA7* negatively regulates ROS accumulation, cell necrosis, and the expression of PTI marker genes and PR genes, functioning as a susceptibility factor during the wheat-*Pst* interaction. Values in this figure are the mean ± SD of three independent replicates. Asterisks indicate significant differences (*P < 0.05, **P < 0.01) relative to the WT plants using the two-tailed Student’s *t*-test

We further used RNA-guided DNA endonucleases (CRISPR-Cas9) as a genome-editing approach (Oliva et al. 2019; Li et al. 2022) to inactivate all three *TaCESA7* homoeologs in the wheat genome. We designed two guide RNAs (gRNAs) for targeting the CDS at the 5′ end of *TaCESA7*−3A, *TaCESA7*−3B, and *TaCESA7*−3D, respectively, based on the conserved sequences among these three gene copies in wheat. *TaCESA7*-knockout (*TaCESA*7 KO) lines were successfully obtained in which all three homoeologs contained deletions or insertions leading to frameshift mutations in the targeted region (Fig. 7A). After inoculation with the predominant *Pst* races CYR31, CYR32, CYR33 and CYR34, *TaCESA7*KO plants exhibited a strong hypersensitive response (HR) with only a few scattered urediniospore pustules detected (Fig. 7B). The fungal biomass of *Pst* was significantly lower in the *TaCESA7*KO plants compared to that on the WT plants (Fig. 7C). These data confirmed that the loss of *TaCESA7* dramatically improves the resistance of wheat to *Pst*. Meanwhile, we monitored the wheat traits of *TaCESA7*KO plants, and the results showed that the increased resistance of TaCESA7 to *Pst* did not compromise the plant height, tiller number, ear length, thousand-grain weight, grain width, and grain length in the field (Fig. 7D-H; Fig. S13 A-C). Additionally, we further evaluated the adult resistance of *TaCESA7*KO plants by inoculation with a mixture of the prevalent *Pst* races in the field trials. The results showed that the *TaCESA7*KO plants with fewer uredia exhibited enhanced resistance compared with WT (Fig. S13D). The disease severity of *TaCESA7*KO plants was significantly decreased (Fig. S13E).

Based on the above experimental results, we concluded that upon infection of *Pst* in wheat, silencing *TaCESA7* leads to a reduction in cellulose synthesis. The decrease in the level of cellulose synthesis can activate lignin synthesis and defense responses, promoting a significant increase in the content of lignin, the accumulation of ROS, cell necrosis, and the expression of PTI marker genes and PR genes, thereby enhancing the resistance of wheat to *Pst* (Fig. 7I). Therefore, *TaCESA7* functions as a susceptibility gene during wheat infection with the stripe rust fungus, and the inactivation of *TaCESA7* does not have an obvious impact on the major agronomic traits of wheat.

## Discussion

*CESA* is a multigene family consisting of more than eight members in higher plants (Suzuki et al. 2006). Since identification of the first *CESA* gene in 1996 (Pear et al. 1996), 10 *CESA* genes have been reported in Arabidopsis (Richmond et al. 2000; Heidari et al. 2019), 11 in rice (Wang et al. 2010, 2012), 16 in maize (Holland et al. 2000; Appenzeller et al. 2004), and 22 in wheat (Kaur et al. 2016). The reason for this much higher number of *CESA* genes in wheat may be related to the fact that wheat is an allohexaploid plant with a complex genome, with approximately 80–90% of its genome consisting of repeat DNA sequences (Consortium, 2014). In this study, we documented 24 *CESA* genes from wheat with three paralogs in the homoeologous genomes A, B, and D (Table S1). Phylogenetic tree analysis showed that *TaCESA7* clustered in the same branch as *OsCESA4* and *AtCESA8* (Fig. S4)*.* The comparison of gene structure and analysis of direct homologs showed that *TaCESA1*, *2*, and *6* and *TaCESA4*, *7*, and *8* are involved in cellulose synthesis of the PCW and SCW in wheat, respectively (Kaur et al. 2016). *TaCESAs* are functionally similar to *AtCESA*s. The 10 *CESA* genes of Arabidopsis can be divided into six categories, including three genes (*AtCESA1*, *AtCESA3,* and *AtCESA6*) that participate in formation of the PCW and three genes (*AtCESA4*, *AtCESA7,* and *AtCESA8*) that participate in formation of the SCW (Endler et al. 2011; Hill et al. 2014).

Plant CESAs, members of the GT2 glycosyltransferase family, catalyze β-glycosidic bonds in cellulose synthesis and function as intrinsic plasma membrane proteins with cytoplasmic catalytic domains (Burton et al. 2010; Desprez et al. 2007). Transmembrane domain analysis predicted eight transmembrane helices in TaCESA7, likely forming a membrane pore for glucan chain extrusion (Kurek et al. 2002). Subcellular localization confirmed its plasma membrane specificity (Fig. 1C, D), aligning with CSC dynamics observed in fluorescently tagged CESAs (Paredez et al. 2006; Wan et al. 2021). CSCs typically assemble from six CESA subunits, with Arabidopsis CESA7/8 forming homodimers and interacting with CESA4 (Li et al. 2013). Split-luciferase assays revealed TaCESA7 self-dimerization and heterodimerization with TaCESA8, but not TaCESA4 (Fig. 1D, E), mirroring preferential subunit interactions critical for CSC assembly (Li et al. 2013; Mansoori et al. 2014). This specificity suggests distinct structural roles: TaCESA7-TaCESA8 dimers may nucleate CSC hexamers, while TaCESA4 integrates via alternative interfaces. Such interaction selectivity implies mechanistic divergence in CSC organization between monocots and dicots (Olek et al. 2014). Collectively, wheat secondary CESAs synthesize cellulose via membrane-localized dimers that hierarchically assemble into functional CSCs, with TaCESA7 acting as a core dimerization hub. These findings advance understanding of CSC architecture and its evolutionary conservation.

Although CESAs are recognized as essential enzymes for plant cell wall formation, their role may well reach beyond the structural aspect of providing a physical barrier (Arya et al. 2021). The Arabidopsis cell wall mutant *CESA3* (*cev1*) exhibits increased resistance to certain pathogens with constitutively activated jasmonic acid (JA)- and ethylene (ET)-mediated defense responses (Ellis et al. 2002). In this study, we identified the *TaCESA7* with significantly up-regulated expression in response to the induction of *Pst* CYR31, the affinity subspecies of stripe rust, in wheat. After overexpression of *TaCESA7*, the spore production level of the fungus increased on the leaves of wheat, along with other obvious symptoms of infection (Fig. 2). Silencing of *TaCESA7* resulted in the rapid induction of up-regulated expression of *PR* genes and ROS accumulation, as well as an effector-triggered immunity (ETI)-associated when challenged with virulent races of *Pst* (Fig. 3; Fig. 4). Transcriptome analysis demonstrated that silencing of *TaCESA7* enhanced the expression of defense-related genes and phenylpropanoid biosynthesis genes (Fig. 5). Moreover, the cellulose content in *TaCESA7*-RNAi plants was reduced by 38–53% (Fig. 6A). It is speculated that cellulose synthase deficiency may trigger the CWI pathway, which subsequently activates stress-responsive transcription factors through a signaling cascade (Desaint et al. 2024). These signals are transduced to the nucleus to modulate the expression of defense-related genes and metabolism-related genes (Zhao et al. 2023). Plants have evolved dedicated mechanisms for maintaining CWI, comprising a diverse set of plasma membrane-resident sensors and pattern recognition receptors that perceive alterations in CWI or wall polysaccharides released by pathogen hydrolases, thereby triggering defense responses (Bacete et al. 2018; Xia et al. 2024). Additionally, reduced consumption of UDP-glucose for cellulose biosynthesis may indirectly affect the expression of genes involved in carbohydrate metabolism and stress responses (Molina et al. 2024).

The plant phenylpropane pathway is involved in disease resistance and immunity since the metabolically synthesized lignin promotes the degree of lignification of cells, forming a physical barrier on the cell wall to prevent the pathogen invasion of cells (Yadav et al. 2020). The change of lignin content in the cell wall significantly changes the resistance/susceptibility phenotype of plants (Miedes et al. 2014). In addition, overexpression of the ligno-suberin pathway genes in tomato resulted in enhanced resistance to *R. solanacearum* by restricting movement of the pathogen (Kashyap et al. 2022; Lee et al. 2019). Our subsequent experiments showed that the SCW thickness did not change significantly in *TaCESA7*-RNAi plants, whereas the lignin content and the degree of lignification increased remarkably (Fig. 6). It is speculated that this is because the inhibition of the *TaCESA7* disrupts the balance between lignin and cellulose synthesis, leading to the accumulation of lignin, which enhances wheat disease resistance (Ma 2024; Hématy et al. 2007). Meanwhile, we also used RT-qPCR to validate the expression levels of key lignin biosynthesis genes in TaCESA7-RNAi lines. The data show significant upregulation of these genes, consistent with the lignin content phenotype (Fig. 6H-M). While this does not establish direct regulation by *TaCESA7*, it strengthens the link between *TaCESA7* loss and lignin pathway activation. This finding indicated that silencing *TaCESA7* led to cell wall remodeling and lignin deposition, which ultimately enhanced the wheat’s resistance to *Pst*.

As the central component of the cell wall, cellulose affects both the growth and defense response of plants (Hu al. 2018). Plant mutants impaired in cellulose synthesis exhibit severe developmental defects such as dwarfing and reduced yield (Li et al. 2018). In this study, we measured the seedling growth and agronomic traits of *TaCESA7*-RNAi and *TaCESA7*KO plants, including the thousand-grain weight, ear length, plant height, grain length, and grain width, indicating no significant difference compared with those of WT plants ((Fig. 7D-H; Fig. S9; Fig. S13 A-C). These results suggested that silencing *TaCESA7* increased wheat resistance but did not affect its agronomic traits. The absence of obvious morphological defects in *TaCESA7*-RNAi and *TaCESA7*KO plants may be attributed to compensatory mechanisms unique to *Poaceae* species. On the one hand, incomplete gene silencing results in residual activity of the CSC. Although *TaCESA7* expression is downregulated, its homologs *TaCESA4* and *TaCESA8* likely maintain sufficient cellulose microfibril crystallinity (Richmond et al. 2000). On the other hand, an increase in the deposition of mixed-linked glucan (MLG) can compensate for the reduced cellulose (Vega-Sánchez et al. 2013). In addition, *TaCESA7* shows tissue-specific silencing, sparing meristematic zones where primary wall *CESAs* (*TaCESA1/3/6*) predominate (Burton et al. 2010). These mechanisms underscore the robustness of cellulose biosynthesis in grasses and provide a framework for understanding how plants balance wall integrity with developmental plasticity. Future studies using transcriptomics, proteomics, and live-cell imaging of CSCs will further clarify these adaptive strategies, enabling precision breeding of wheat with tailored cell wall traits.

## Conclusion

In summary, our study indicates that *TaCESA7* functions as a susceptibility gene in the wheat-*Pst* interaction. During *Pst* infection, the knockdown of *TaCESA7* disrupts the assembly of CSCs on the plasma membrane, impairs SCW cellulose biosynthesis, and ultimately results in reduced cellulose synthesis. This structural weakening triggers compensatory activation of phenylpropanoid biosynthesis and plant-pathogen interaction pathways, thereby driving lignin deposition to reinforce cell walls and restrict pathogen spread. Meanwhile, reduced cellulose synthesis may release cell wall-derived DAMPs, thus initiating PTI. Additionally, the accumulation of ROS and significant increase in cell necrosis area also enhance wheat resistance to *Pst*. The plant cell wall is not only a structural passive defense barrier, but also a dynamic active defense system. Therefore, cell wall mediated resistance is an important component of the plant immune response system (Hardham et al. 2007). Through the functional analysis of cell wall-related genes, this study helps to elucidate the mechanism of cell wall-mediated resistance, and further enrich the understanding of plant immune system and its action mechanism. However, its in-depth molecular interaction mechanism still needs further research.

## Materials and methods

### Plant materials and strains

The wheat (*Triticum aestivum* L.) Suwon 11 carrying the disease resistance gene *YrSu* was used for evaluation of wheat expression patterns, wheat protoplasts generation, and VIGS. The wheat cultivar Fielder was used to establish the *TaCESA7*-RNAi lines, *TaCESA7*KO mutant, and *TaCESA7-*OE lines. Wheat seedlings were grown in a growth room at 16℃ with 16 h of light and at 14℃ with 8 h of dark. The *Pst* race CYR23 was propagated on the Mingxian169 wheat variety, whereas the other rust fungal races CYR31, CYR32, and CYR34 were grown on Su11 wheat. The Su11 cultivar formed an affinity interaction system with CYR31, CYR32, and CYR34 and a non-affinity interaction system with CYR23. The *Pst*-inoculated wheat plants were incubated at 16℃ for 24 h in the dark as described previously (Guo et al. 2022). The light intensity was 20,000 lx. *N. benthamiana* was grown at 23℃ under a 16 h light/8 h dark photoperiod for the tobacco subcellular localization assays. For transformation, the *Escherichia coli* strain DH5α and the *Agrobacterium* strains GV3101 and EH105 were used.

### RT-qPCR analysis

Gene expression levels were determined by RT-qPCR using the SYBR Green method on a CFX-conjugated real-time PCR detection system (Bio-Rad, Hercules, CA, USA). RT-qPCR analyses were performed using data from three samples with each group containing three technical replicates, and the data were analyzed using the comparative 2^−ΔΔCT^ method (Livak et al. 2001; Yuan et al. 2021). All of the primers used are listed in Table S2.

### Quantification of TaCESA7 expression level after Pst and PAMP treatment

To detect the expression level of *TaCESA7* under the treatment of *Pst*, wheat eaves were collected at 0, 12, 24, 48, 72, and 120 hpi for RNA isolation (Guo et al. 2023). The detection of PAMP-induced gene expression was conducted as previously described (Guo et al. 2022). Wheat leaves were treated with 10 μM chitin (Santa Cruz. Biotechnology) for 10, 30, 60, or 180 min, then were performed by RT-qPCR assays. The primers listed in Table S2.

### Cloning of TaCESA7 and sequence analysis

To clone the sequence of *TaCESA7*, we designed specific primers using Primer version 5.0 software (Table S2). The resulting sequences were compared using CS genome data from the International Wheat Genome Sequencing Consortium (https://urgi.versailles.inra.fr/blast) and Ensembl Plants (http://plants.ensembl.org). Multiple sequence comparison was performed using DNAMAN version 8 software. The transmembrane structural domain of TaCESA7 was identified using TMHMM (http://www.cbs.dtu.dk/services/TMHMM/). We constructed the phylogenetic tree by MEGA 7 through the neighbor-joining method with a bootstrap option of *n* = 1000 and the pairwise deletion of gaps (Kumar et al. 2016a, b).

### Plasmid construction

For the construction of subcellularly localized vectors, the coding sequence of *TaCESA7* without a stop codon was amplified and inserted into the *Nco*I and *Spe*I cleavage sites, respectively, of the plant binary expression vector pCAMBIA1302, which contains a GFP-tag sequence. For split-luciferase assays, the CDSs of *TaCESA4*, *TaCESA7*, and *TaCESA8* were subcloned into the C- or N-terminal fragment of LUC (cLUC or nLUC) with *Bam*HI and *Sa*lI restriction sites, respectively. To overexpress *TaCESA7* in wheat plants, we used *Spe*I to construct *TaCESA7*−3HA in the pCUB plant overexpression vector. The open reading frame of *TaCESA7* was completely inserted into the pCUB overexpression vector driven by the maize (*Zea mays*) ubiquitin promoter. To create RNAi transgenic materials, we designed RNAi-specific primers containing attB1 and attB2 sites by RNAi silencing technology, constructed *TaCESA7* in the intermediate vector pDONR221 using the BP reaction, and finally constructed the PC336 vector using the LR reaction, which ultimately yielded the construct PC336-*TaCESA7*. For plasmid construction of silencing systems, based on a BLASTN search of the National Center for Biotechnology Information database (http://www.ncbi.nlm.nih.gov/), two cDNA fragments were derived from the CDS (200 bp, nucleotides 251–451; 209 bp, nucleotides 1043–1252) with the lowest sequence similarity to other wheat genes among other wheat families, which were analyzed by PCR. The two cDNA fragments were amplified, and the recombinant plasmids *TaCESA7*−1as and *TaCESA7*−2as were antisense-constructed and inserted into the *Pac*I and *Not*I cleavage sites of the viral plasmid γ, respectively (Holzberg et al. 2002).

### Subcellular localization of TaCESA7

To determine the subcellular localization of TaCESA7, we constructed the TaCESA7-PGFP16318 recombinant vector for the transient transformation of wheat protoplasts. To further validate the specificity of the localization of TaCESA7, the recombinant plasmid TaCESA7-GFP was transformed with *Agrobacterium* and then injected into tobacco. TaWpi6-mCherry was used as the positive control localized to the plasma membrane (Zhang et al. 2023). An FV3000 confocal microscope (Olympus, Tokyo, Japan) was used to observe the fluorescence signals under the following conditions: 488 nm laser wavelength and 500–540 nm detection wavelength for GFP; 561 nm laser wavelength and 570–670 nm detection wavelength for mCherry; 640 nm laser wavelength and 650–750 nm detection wavelength for chloroplasts. All assays were repeated more than three times and showed reproducible results.

### Split-luciferase assays

Split-luciferase analysis was performed as previously described (Guo et al. 2022). In brief, the vectors TaCESA7-nLUC and TaCESA4/TaCESA7/TaCESA8-cLUC were co-injected in tobacco leaves. GUS-cLUC and GUS-nLUC were used as negative controls. Subsequently, 1 mM luciferin (AbMole) was applied to the injected area at 48 hpi and the LUC activity was detected by the PlantView100 system.

### VIGS

In the VIGS assay, to silence *TaCESA7*, two segments of 100–300 bp within the conserved region of the gene were selected as silencing segments, and then the recombinant plasmids BSMV:Ta*CESA7* were constructed as described previously (Holzberg et al. 2002; Gan et al. 2024). At 14 dpi, the fourth leaf of wheat plants was inoculated with *Pst* CYR31 and maintained at 16℃. The *Pst*-infected leaves were sampled at 0, 24, 48, and 120 hpi for silencing efficiency and histological observation. Changes in the fungal biomass of *Pst*-infected leaves were detected at 5 and 14 dpi as described previously (Lee et al. 2008). Quantification of the wheat stripe rust elongation factor gene *PsEF* against stripe rust fungi was performed using *TaEF* as an endogenous reference gene for normalization (Yin et al. 2009). The phenotypes of wheat leaves were observed 14 dpi. The VIGS experiments were repeated at least three times.

### Generation and identification of transgenic wheat plants

To evaluate the possible role of *TaCESA7* in the wheat-*Pst* interaction, we cloned the CDS of *TaCESA7* into the pCUB plant overexpression vector based on the Fielder wheat cultivar to obtain transgenic overexpression wheat plants. We also constructed *TaCESA7* on the PC336 vector by the RNAi silencing technique using BP and LR reactions, and then transformed the wheat healing tissue culture after transferring into *Agrobacterium* EHA105 receptor cells to obtain *TaCESA7*-RNAi plants. Genomic DNA was extracted by the CTAB method, and overexpression- or RNAi-positive plants were identified by PCR with ddH_2_O as a blank control and WT plants as a negative control.

### Histological observations of fungal growth

For observation of hyphal development, *Pst*-infected leaves were stained by wheatgerm agglutinin (WGA) conjugated to Alexa-488 (Amresco, USA) as previously described (Ayliffe et al. 2011). The *Pst* fungal substomatal vesicle, hyphal length, and hyphal infection areas were observed with an Olympus BX-53 microscope (Olympus, Tokyo, Japan). Cell death staining was performed with trypan blue as described previously (Peterhansel et al. 1997), in which leaves inoculated with *Pst* were stained in a 0.5% solution of trypan blue in lactophenol for 2 days at room temperature. H_2_O_2_ accumulation was visualized by a DAB (Sigma-Aldrich, St. Louis, MO, USA) staining assay as previously described (Guo et al. 2022). The cleared and stained segments were observed and photographed by epifluorescence microscopy (Olympus, Tokyo, Japan). Fifty infection sites were counted at each time point. Three independent biological replicates were performed.

### Knockout of TaCESA7 by the CRISPR-Cas9 system

To knock out *TaCESA7* through CRISPR-Cas9 gene editing, two gRNA target sites (named Target 1 and Target 2) for *TaCESA7* on the conserved sequences of the wheat A, B, and D genomes were designed via the E-CRISP website (http://www.ecrisp.org). The two designed gRNAs targeting *TaCESA7* were cloned into the VK005-6 CRISPR-Cas9 vector (Wang et al. 2022) using the Plant Cas9/gRNA Plasmid Construction kit (Viewsolid Biotech). The knockout vector VK005-6-*TaCESA7* was then transformed into wheat using the *Agrobacterium-*mediated wheat transformation system. The transgenic plants were validated by a PCR assay for detection of the inserted Cas9 gene. To further analyze the positive transgenic plants, genome-specific primers were designed to amplify the target regions of the A, B, and D genomes (Table S2). The PCR products were sequenced to identify the *TaCESA7* mutant plants. To determine the genotype of *TaCESA7* mutant plants, PCR products were cloned into the pMD-18 T vector (Takara Biotech) followed by sequencing.

### Safranine and solid green double staining

Safranine and solid green double staining was performed as described previously (Gao et al. 2019). The leaves of *TaCESA7*-RNAi plants in 48 hpi were paraffin-sliced, dewaxed, hydrated, and stained with saffranine for 2 h. The samples were gently washed in running water and then subject to solid green staining for 30–60 s. The sections were then treated with anhydrous ethanol for 2 min and anhydrous ethanol for 2 min respectively, followed by destaining with xylene I for 1 min and xylene II for 2 min. Finally, the sections were sealed with neutral gum and examined under the microscope.

### *PAL, cellulose, lignin, and H*_*2*_*O*_*2*_* content detection*

At 24 hpi, 0.1 g leaves of Fielder and *TaCESA7*-RNAi transgenic wheat were collected for determination of PAL activity with the Micro Phenylalanine Ammonia-lyase (PAL) Assay Kit (Solarbio; BC0215), cellulose content with the Cellulose (CLL) Content Assay Kit (Solarbio; BC4280), and lignin content with the Lignin Content Assay kit (Solarbio; BC4205), according to the manufacturer instructions. H_2_O_2_ content of *TaCESA7*-RNAi transgenic wheat was detected by the plant H_2_O_2_ content detection kit (Solarbio, BC3590) according to the manufacturer’s instructions. The experiments were repeated at least three times.

### RNA-seq analysis

To analyze global gene expression changes of *TaCESA7*-RNAi plants after *Pst* infection, UID-mRNA sequencing was carried out using RNA samples extracted from *TaCESA7*-RNAi and WT (wheat cv. Fielder) seedlings at 24 hpi with CYR31. The RNA-seq assay was conducted by the Novogene Bioinformatics Technologies Co. Ltd. Each sample generated 6G data and produced paired-end reads, and they were mapped on the Ensembl *Triticum aestivum* genome using HISAT2. The National Center for Biotechnology Information database serves as the foundation for both functional categorization and genome annotation (Kim et al. 2019).

### Statistical analysis

The data are based on three biological replicates and were statistically analyzed using two-tailed Student’s *t*-test, with *P < 0.05, **P < 0.01, and ns indicating statistically significant, highly significant differences, and no statistical significance, respectively. Detailed *P*-values are shown in Supplemental Table S4. All analyses were performed using GraphPad Prism 8.0.

### Accession numbers

The Ensembl Plants database was used to search the genome sequence data of this paper, with accession numbers as follows: Ta*CESA7 A* (TraesCS3 A02G289900), Ta*CESA7B* (TraesCS3B02G324500) and *TaCESA7D* (TraesCS3D02G289600).

## Supplementary Information

Supplementary Material 1.

Supplementary Material 2.

Supplementary Material 3.

Supplementary Material 4.

Supplementary Material 5.

## Data Availability

All data generated or analyzed during this study are included in this published article and its supplementary information files.

## Abbreviations

CESAs: Cellulose synthases
CSC: Cellulose synthase complex
CSL: Cellulose synthase-like protein
CWI: Cell wall integrity
DAB: 3,3′-Diaminobezidine
DAMPs: Damage-associated molecular patterns
dpi: Days post-infection
DEGs: Differentially expressed genes
ETI: Effector-triggered immunity
hpi: Hours post-infection
HR: Hypersensitive response
KEGG: Kyoto Encyclopedia of Genes and Genomes
*N. benthamiana*: *Nicotiana benthamiana*
PAL: Phenylalanine ammonia-lyase
PAMP: Pathogen-associated molecular pattern
PCW: Primary cell wall
*Pst*: *Puccinia striiformis* f. sp. *tritici*
PTI: PAMP triggered immunity
ROS: Reactive oxygen species
RT-qPCR: Reverse transcription-quantitative polymerase chain reaction
SCW: Secondary cell wall
VIGS: Virus-induced gene silencing
