# Supplementary material for: Cellulose synthase TaCESA7 negatively regulates wheat resistance to stripe rust by reducing cell wall lignification
Source: Stress Biol. 2025 Jun 16;5(1):42. doi: 10.1007/s44154-025-00244-7 (PMC12170496; doi:10.1007/s44154-025-00244-7)
Supplement: Supplementary file 1 — Supplementary Material 1. [file 44154_2025_244_MOESM1_ESM.docx]

**Supporting Information**

Article title: **Cellulose synthase *TaCESA7* negatively regulates wheat resistance to stripe rust by reducing lignin biosynthesis**

Author: Yanqin Zhang, Longhui Yu, Shuangyuan Guo,Xueling Huang, Yihan Chen, Pengfei Gan, Yi lin, Xiaojie Wang, Zhensheng Kang, and Xinmei Zhang

**Fig. S1** Relative transcript levels of *TaCESAs* in wheat leaves in response to Pst infection.

**Fig. S2** Relative transcript levels of *TaCESA7A*, *TaCESA7B*, and *TaCESA7D* in wheat leaves in response to *Pst* infection.

**Fig. S3** Sequence analysis and gene cloning of *TaCESA7*.

**Fig. S4** Phylogenetic analysis of deduced amino acid sequences of *TaCESAs* from wheat, rice and *Arabidopsis*.

**Fig. S5** Subcellular localization of TaCESA8 and TaCESA4 and non-interaction of TaCESA7 with TaCESA4.

**Fig. S6** PCR and transcript expression analysis of *TaCESA7* overexpression (OE) transgenic plants.

**Fig. S7** Histological observation of Pst development in leaves from *TaCESA7*-OE and WT plants.

**Fig. S8** Silencing *TaCESA7* reduces wheat susceptibility to *Pst* race CYR31 by VIGS.

**Fig. S9** Agronomic traits of *TaCESA7*-RNAi and WT plants in the field.

**Fig. S10** Differentially expressed genes in *TaCESA7*-RNAi transcriptome data.

**Fig. S11** GO enrichment analysis of differentially expressed genes (DEGs) c in the *TaCESA7*-RNAi transgenic lines based on up-regulated genes.

**Fig. S12** The number of differentially expressed genes (DEGs) contained in each KEGG pathway in the *TaCESA7*-RNAi transgenic lines based on down-regulated genes.

**Fig. S13** the agronomic traits and disease phenotypes inoculated with Pst of TaCESA7KO and WT plants in the field.

**Table. S1** The Ensembl IDs of all identified *CESA* genes in wheat, rice and *Arabidopsis*.

**Table. S2** Information of primers used in this study.

**Table. S3** Significantly up- or down-regulated genes in the TaCESA7-RNAi plants.

**Table. S4** Statistical analysis results for each figure.

**
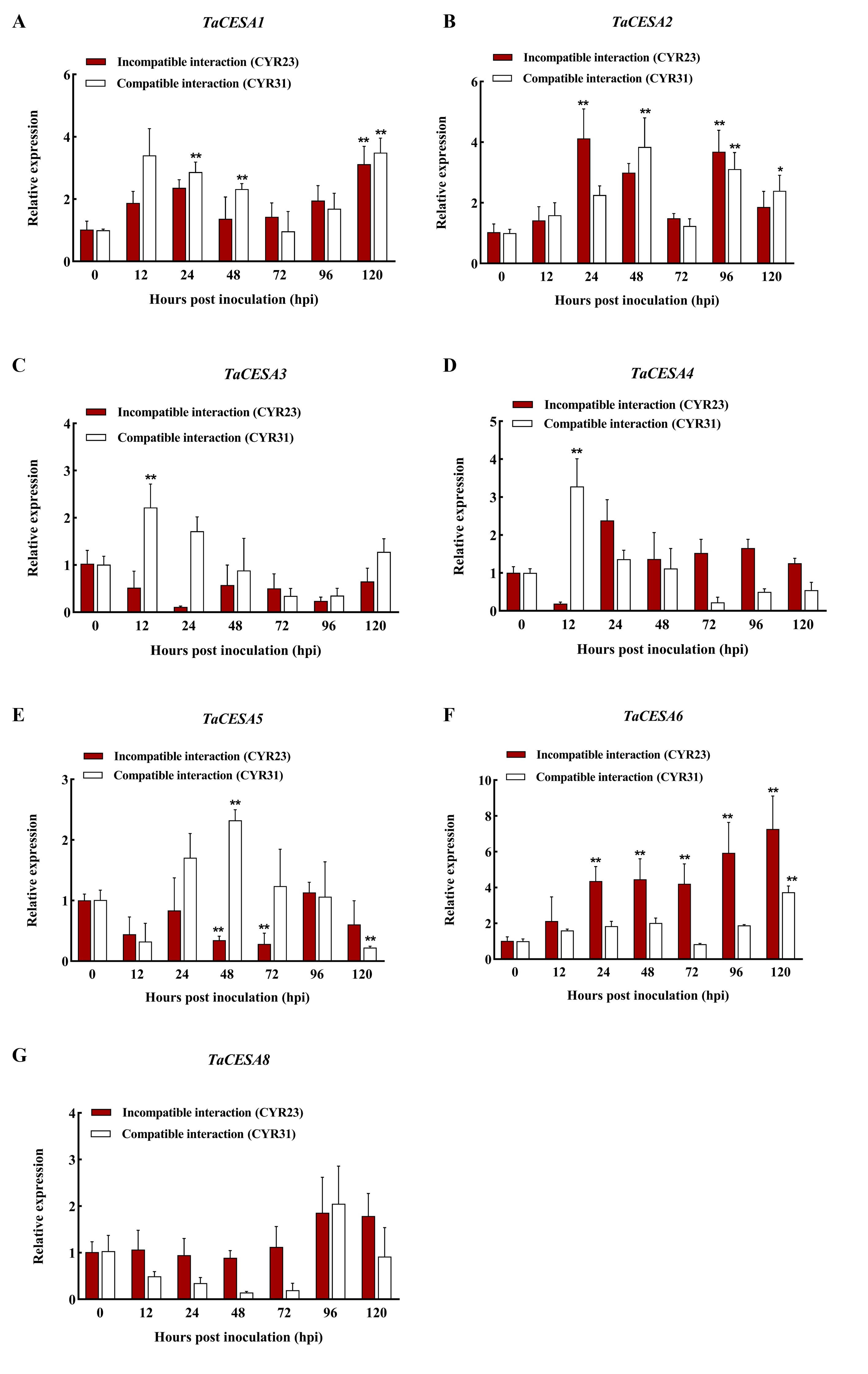
**

**Fig. S1** Relative transcript levels of *TaCESAs* in wheat leaves in response to *Pst* infection. Wheat leaves were sampled for CYR23 and CYR31-inoculated plants at 0, 12, 24, 48, 72, 96, and 120 hpi. Untreated leaves act as control. (**A-G)** The relative expressions of *TaCESA1* **(A)**, *TaCESA2* **(B)**, *TaCESA3* **(C)**, *TaCESA4* **(D)**, *TaCESA5* **(E)**, *TaCESA6* **(F)**, and *TaCESA8* **(G)** were calculated using the comparative threshold method (2^-△△CT^). Expression levels were normalized to *TaEF*. Error bars represent the variation among three independent replicates. Asterisks indicate that the difference in relative expression of *TaCESAs* at that time point was significant (**P* < 0.05, ***P* < 0.01).


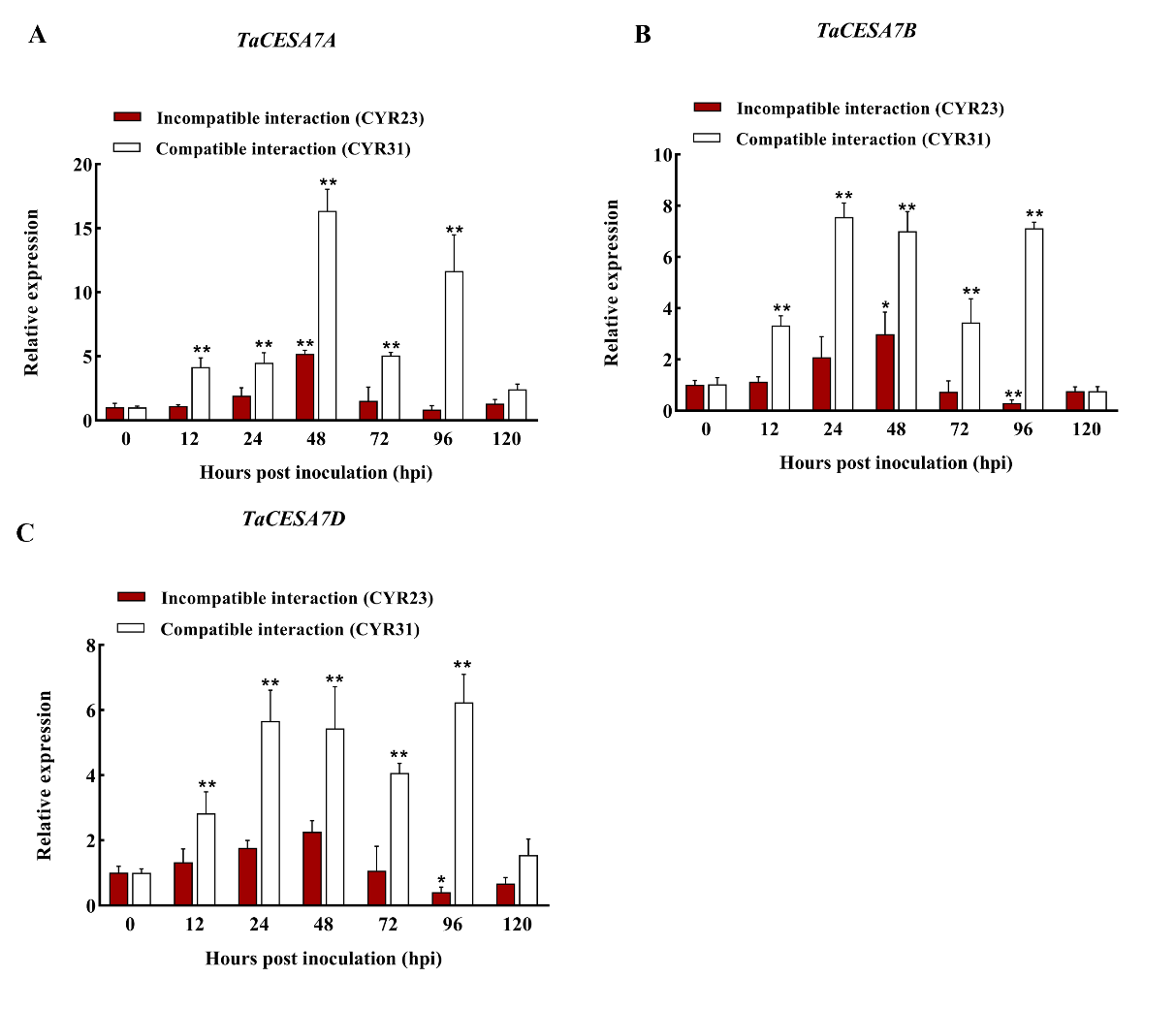


**Fig. S2** Relative transcript levels of *TaCESA7A* **(A)**, *TaCESA7B* **(B)**, and *TaCESA7D* **(C)** in wheat leaves in response to *Pst* infection. Wheat leaves were sampled for CYR23 and CYR31-inoculated plants at 0, 12, 24, 48, 72, 96, and 120 hpi. Untreated leaves act as control. The relative expressions of *TaCESA7A*, *TaCESA7B*, and *TaCESA7D* were calculated using the comparative threshold method (2^-△△CT^). Expression levels were normalized to *TaEF*. Error bars represent the variation among three independent replicates. Asterisks indicate that the difference in relative expression of *TaCESA7A/B/D* at that time point was significant (**P* < 0.05, ***P* < 0.01).

**
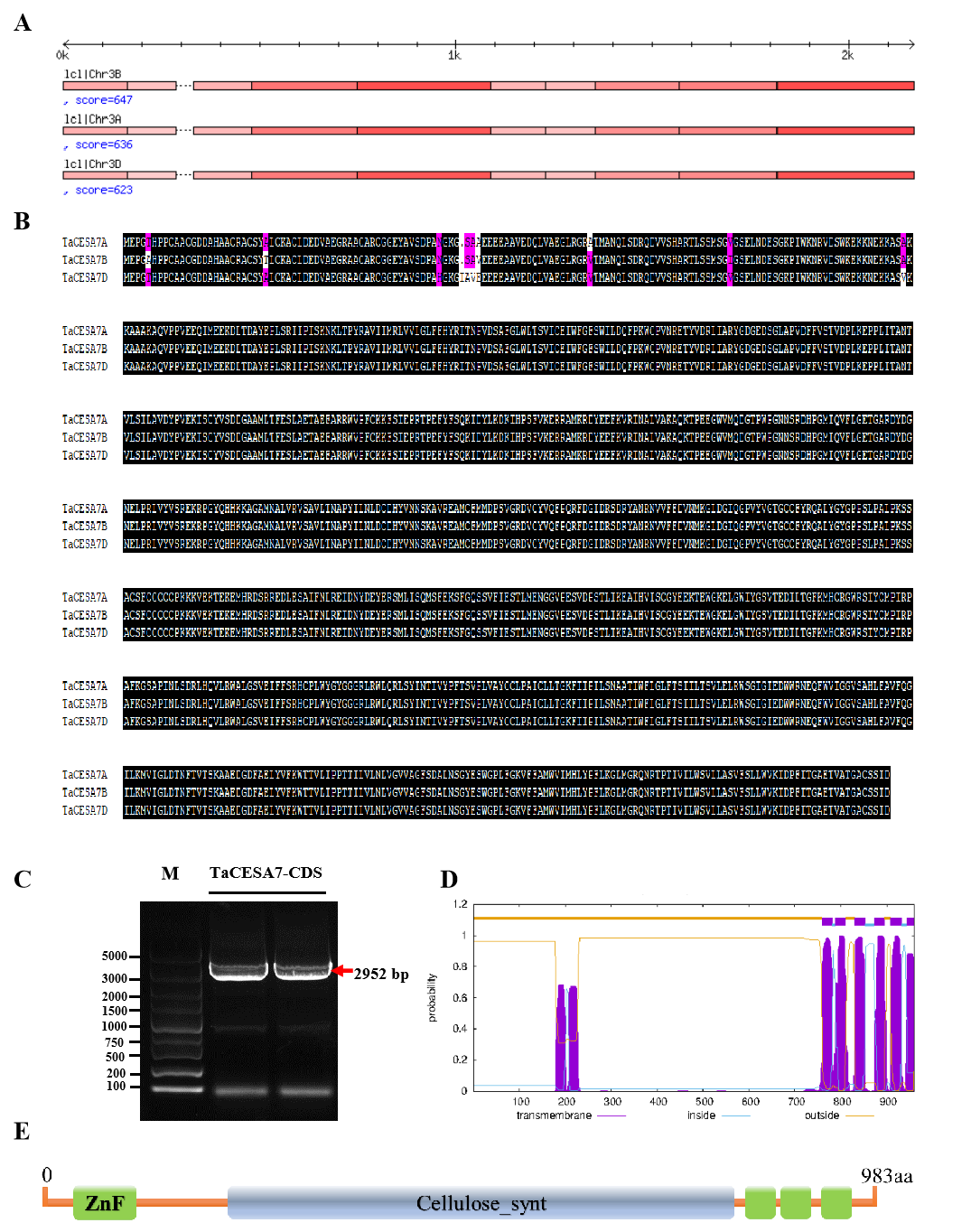
****Fig. S3** Sequence analysis and gene cloning of *TaCESA7*. **(A)** WHEAT URGI online website (https://wheat-urgi.versailles.inra.fr/Projects/IWGSC) analyzed *TaCESA7* on chromosomes 3A, 3B and 3D. **(B)** Amino acid sequence alignment of TaCESA7A, TaCESA7B and TaCESA7D. **(C)** Agarose gel electrophoresis for the amplified products of *TaCESA7* gene. M, DL5000 DNA marker. **(D)** The transmembrane domain of TaCESA7 was identified by using TMHMM Server v. 2.0 (<http://www.cbs.dtu.dk/services/TMHMM/>). **(E)** Conserved domain analysis for TaCESA7 by using SMART (<http://smart.embl-heidelberg.de/>).


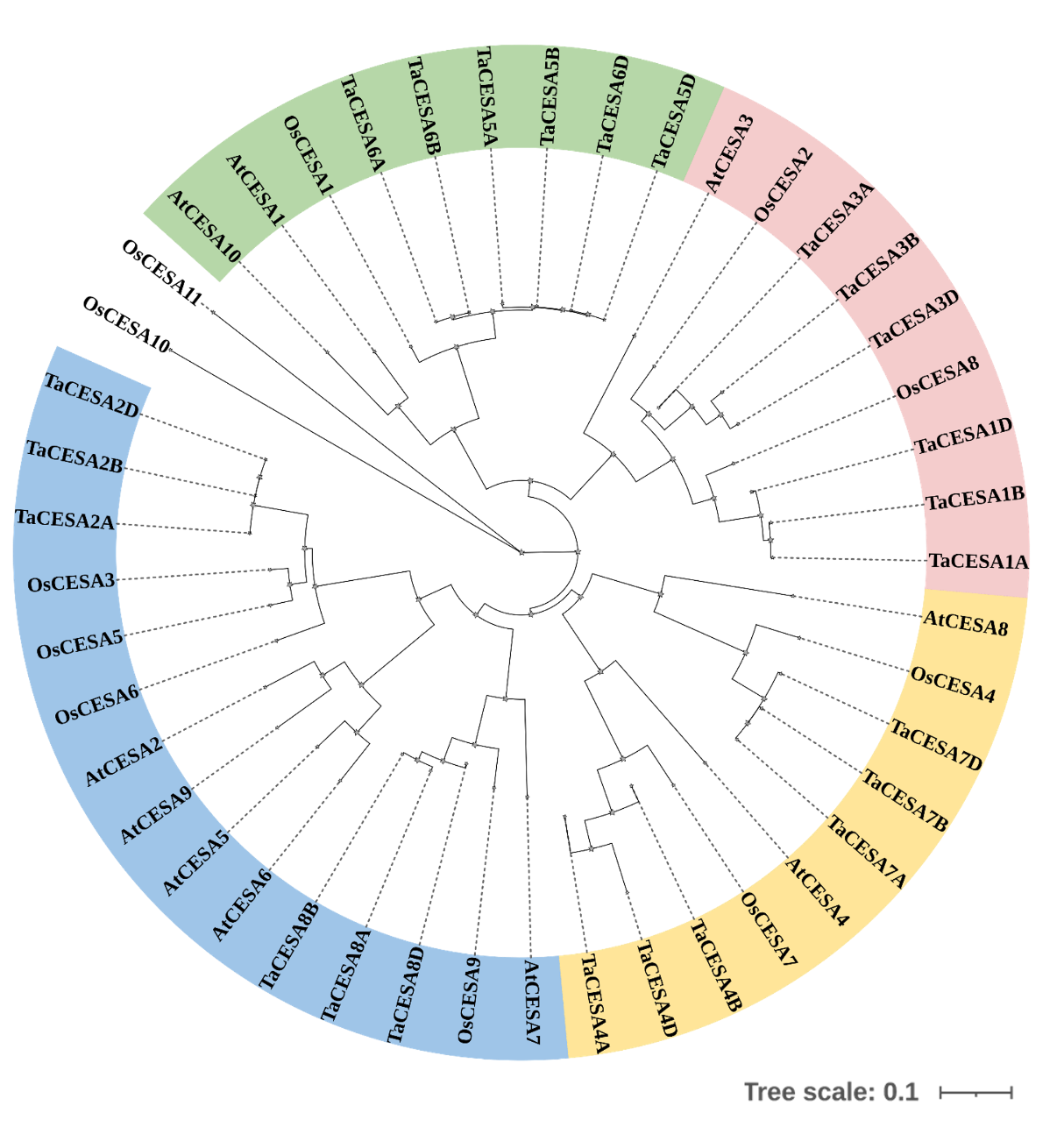


**Fig. S4** Phylogenetic analysis of deduced amino acid sequences of TaCESAs from wheat, rice and *Arabidopsis*. The neighbor-joining tree was created using MEGA7.0 software. The number above the internal branches indicates bootstrap values estimated based on 1000 replications. The scale bar indicates 0.1 substitutions per nucleotide position. Ta, *Triticum aestivum*. Os, *Oryza sativa*. At, *Arabidopsis thaliana*.

**(D)** Localization analysis of TaCESA7 in *N. benthamiana* leaves. TaWpi6-mCherry as a marker of the plasma membrane. All signals are monitored using confocal microscope FV3000. Scale bars = 20μm.


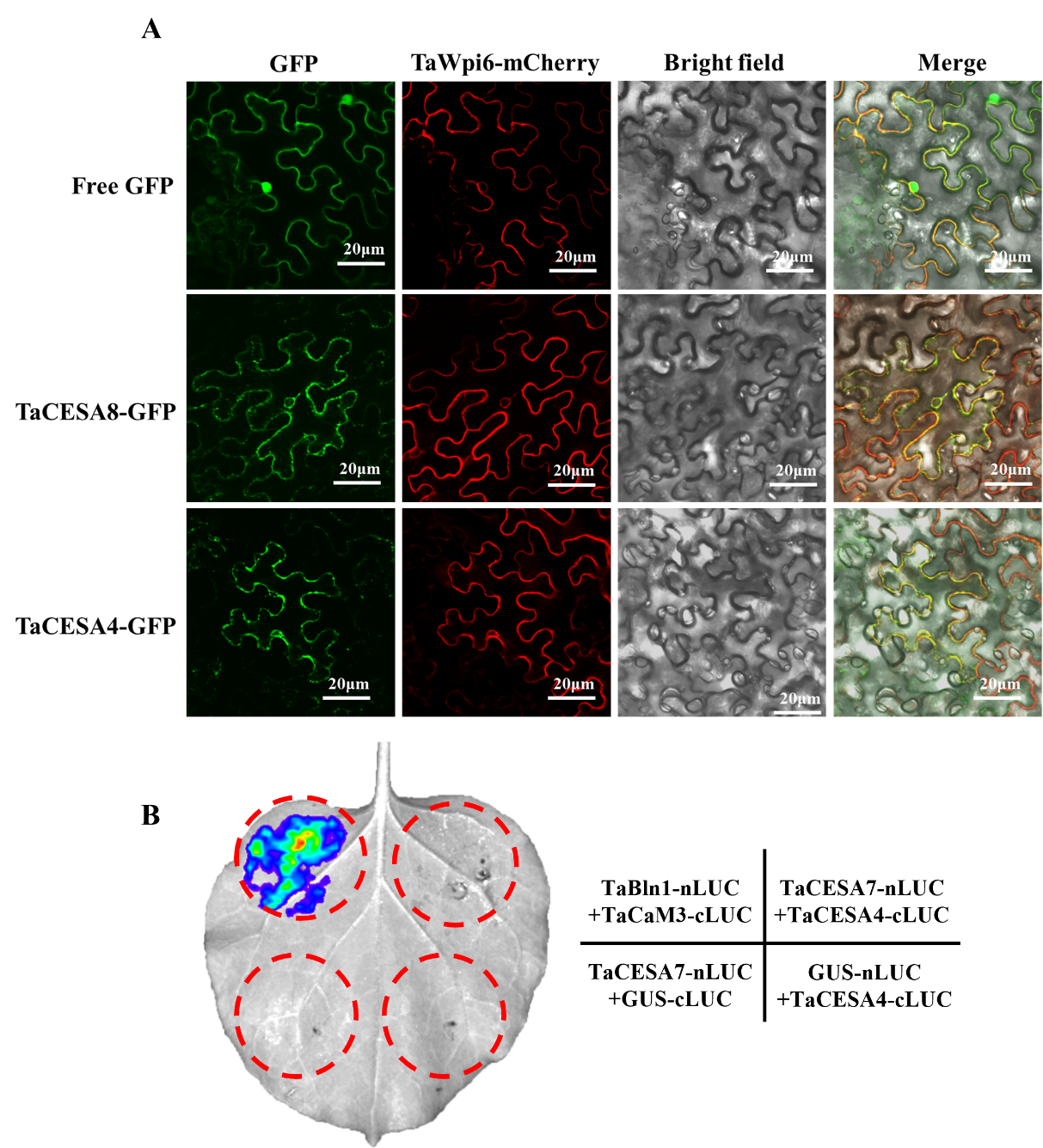


**Fig. S5** Subcellular localization of TaCESA8 and TaCESA4 and non-interaction of TaCESA7 with TaCESA4. **(A)** Co-expression of TaCESA4-GFP or TaCESA8-GFP and TaWpi6-mCherry in *N. benthamiana* leaves. GFP fluorescence is green; TaWpi6-mCherry (red fluorescence) indicates TaWpi6 labeling the plasma membrane. All of the signals were monitored by confocal microscopy. Bar = 20 μm. **(B)** TaCESA7 does not interact with TaCESA4 by firefly luciferase complementation imaging assay. TaBln1-nLUC and TaCaM3-cLUC was used as positive control. The GUS-nLUC was used as negative control.


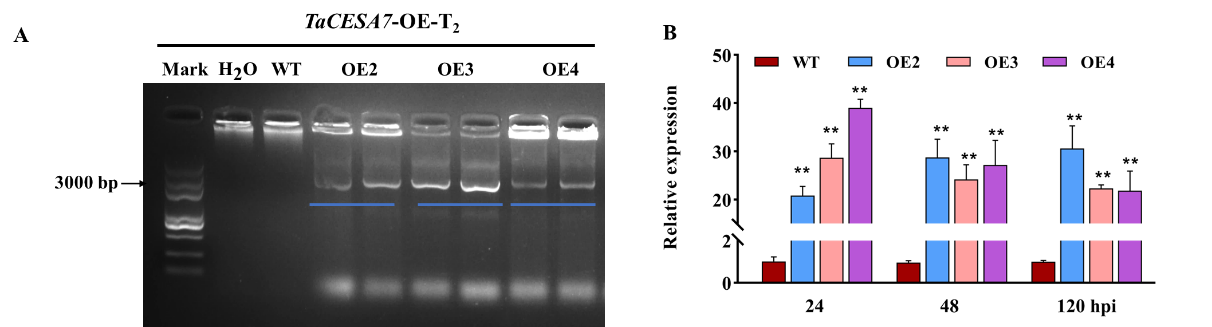


**Fig. S6** PCR and transcript expression analysis of *TaCESA7* overexpression (OE) transgenic plants. **(A)** Transgenic plants of T_2_ generation were analyzed by genomic qPCR for the presence of *TaCESA7* with the primers pCUB-F/pCUB-R. M, DL5000 DNA marker; WT, wild type (Fielder); OE2, OE3, and OE4, *TaCESA7*-OE wheat lines. **(B)** The transcript levels of *TaCESA7* in the transgenic overexpression lines and WT at 24, 48, and 120 hpi with *Pst*. Values are the mean ± SD of three independent replicates. Asterisks indicate significant difference (***P* < 0.01).

**
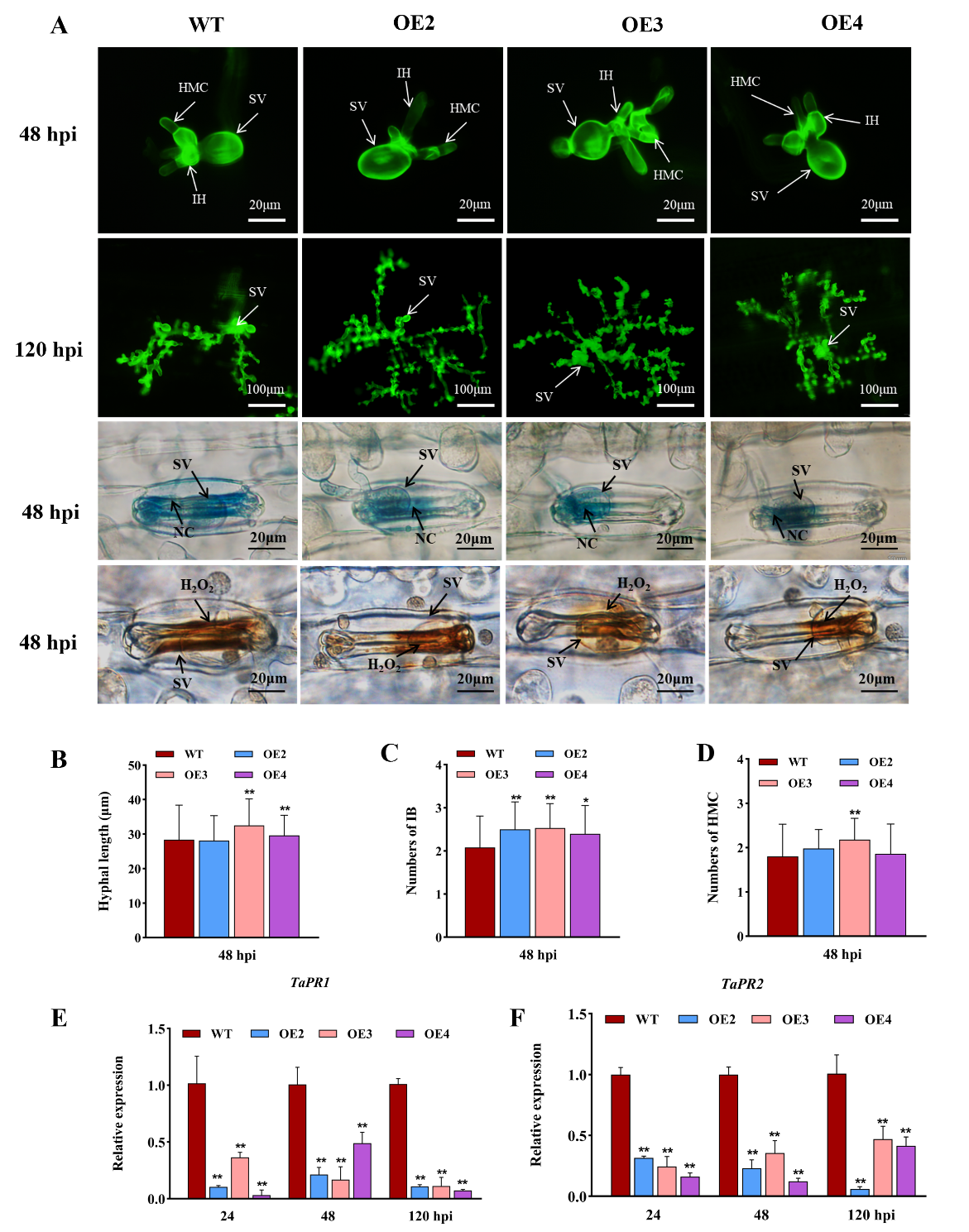
Fig. S7** Histological observation of *Pst* development in leaves from *TaCESA7*-OE and WT plants. **(A)** Fungal structures were visualized with WGA-488 staining and observed by fluorescence microscope at 48 and 120 hpi; Representative pictures of necrotic cell death in *Pst* infected wheat cells detected by trypan blue staining in *TaCESA7*-OE lines at 48 hpi; Representative pictures of H_2_O_2_ accumulation by DAB staining in wheat leaves at 48 hpi after infection with CYR23. SV, substomatal vesicle; HMC, haustorial mother cell; IH, infection hypha. NC, necrotic cell. Bar of 48 hpi = 20 μm, Bar of 120 hpi =100 μm. **(B-D)** The average number of hyphal lengths **(B)**, hyphal branches (HB) **(C)** and haustorial mother cell (HMC) **(D)** of *Pst* in each infection site at 48 hpi. Values were derived from three biological repetitions (50 infection sites each time). **(E-F)** Expression profiles of two pathogenesis-related proteins *TaPR1* **(E)** and *TaPR2* **(F)** were assessed in *TaCESA7*-OE plants compared with the WT plants. The data were normalized to the *TaEF* gene. **B-F,** Values are the mean ± SD of three independent replicates. Asterisks indicate significant differences (**P <* 0.05, ***P* < 0.01)
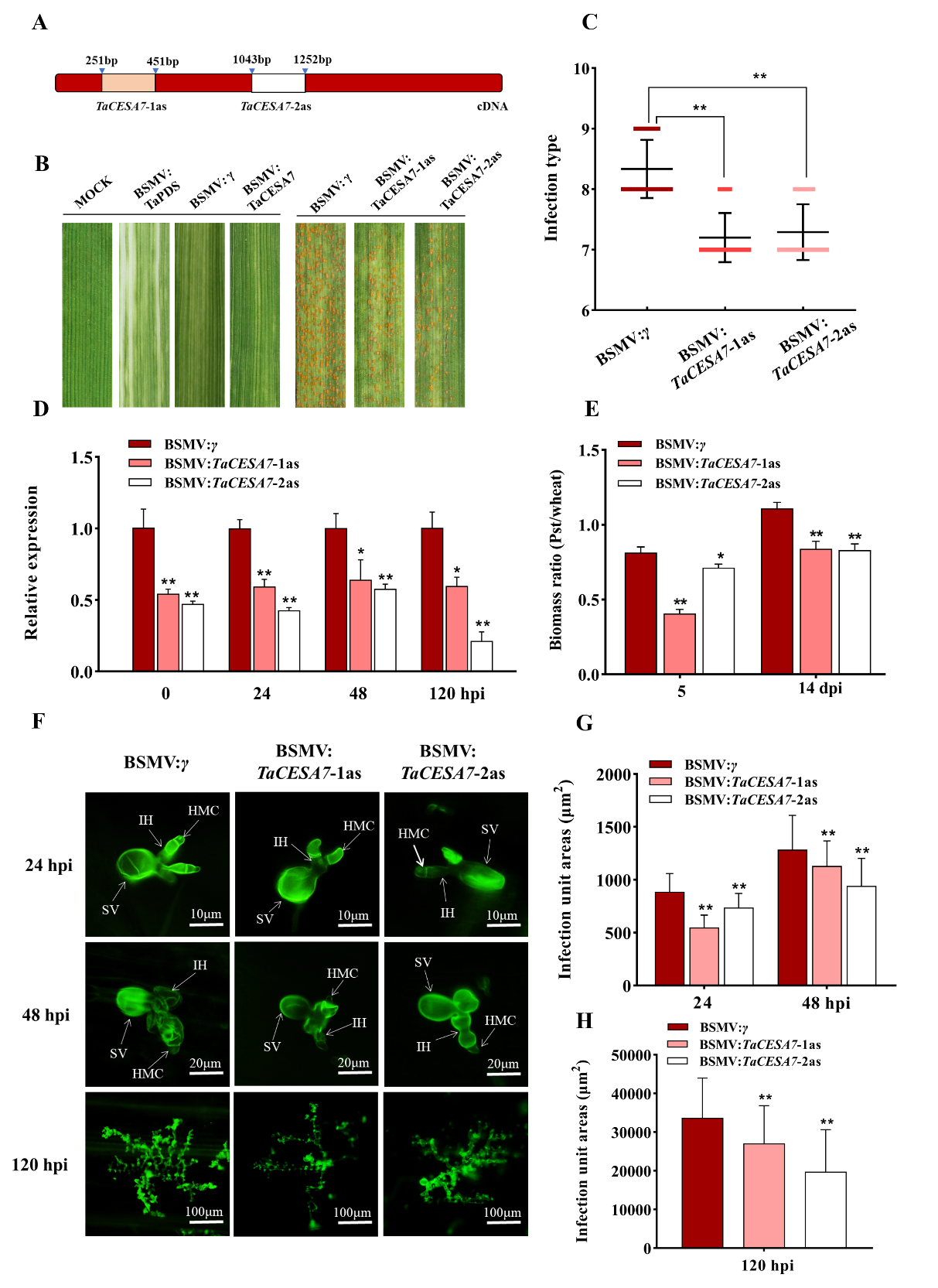
relative to the WT plants using the Student’s *t*-test.

**Fig. S8** Silencing *TaCESA7* reduces wheat susceptibility to *Pst* race CYR31 by VIGS. **(A)** Schematic view of the VIGS construct design about *TaCESA7*. **(B)** Disease phenotype of wheat leaves with silenced *TaCESA7* at 14 dpi with the *Pst* CYR31. The phenotype did not change in wheat leaves mock inoculated. Photobleaching was visible on wheat leaves inoculated with BSMV:*TaPDS*. **(C)** The resistance grade statistics to *Pst* in wheat at 14 dpi. **(D)** The relative expression levels of *TaCESA7* in wheat leaves inoculated with CYR31 were assayed by RT-qPCR at 0, 24, 48 and 120 hpi. **(E)** Biomass ratio of the *Pst*/wheat was assayed by qPCR with total DNA extracted from BSMV-treated wheat leaves infected by CYR31 at 5 and 14 dpi. **(F)** Fungal structures in wheat leaves infected with BSMV and *Pst* were visualized with WGA and the fungal structures were observed under a fluorescence microscope. SV, substomatal vesicle; HMC, haustorial mother cell; IH, infection hypha. Bar of 24 hpi =10 μm, Bar of 48 hpi =20 μm; Bar of 120 hpi =100 μm. **(G)** The infection area of *Pst* in each infection site were counted at 24 and 48 hpi. **(H)** The infection area of *Pst* in *TaCESA7*-silenced plants at 120 hpi. Means were calculated from 50 infection sites. Three independent biological replications were performed. Asterisks indicate a significant difference (**P* < 0.05, ***P* < 0.01) from BSMV:*γ* inoculated plants using the Student’s *t*-test.

**Fig. S9** Agronomic traits of *TaCESA7*-RNAi and WT plants in the field. **(A)** Transgenic plants were analyzed by genomic PCR for the presence of the selected *TaCESA7*-silenced fragment in the RNAi cassette with the primers PC336-F/PC336-R. M, DL2000 DNA marker; WT, wild type (Fielder). **(B)** The growth phenotype of *TaCESA7*-RNAi T_2_ transgenic positive strains and WT plants in the field. **(C)** Wheat seeds from *TaCESA7*-RNAi transgenic and WT plants at the mature stage. **(D-I)** Comparison of six traits in WT and *TaCESA7*-RNAi plants growing in the field: **(D)** plant height (n = 35), **(E)** ear length (n = 35), **(F)** tiller number (n = 35), **(G)** thousand-grain weight (n = 35), **(H)** grain width (n = 35), and **(I)** grain length. Values in this figure are the mean ± SD of three independent replicates. Statistical significance was determined by two-tailed Student’s *t* test.

**
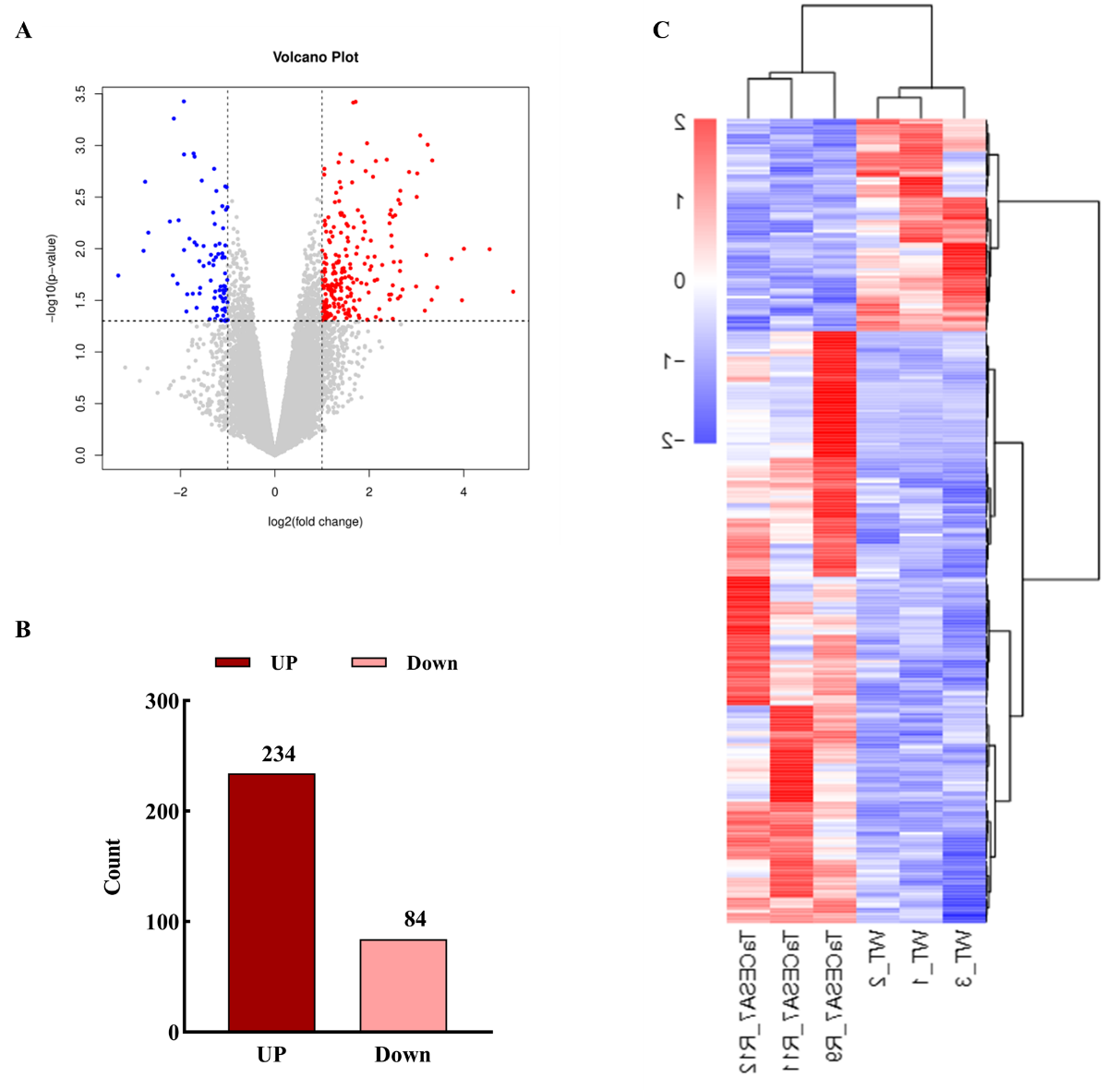
**

**Fig. S10** Differentially expressed genes in *TaCESA7*-RNAi transcriptome data. **(A)** Volcano plot of differentially expressed genes in *TaCESA7*-RNAi transcriptome data. Grey dots represent genes that have not undergone differential expression, blue dots represent genes that have undergone differential down-regulation, and red dots represent genes that have undergone differential up-regulation. **(B)** Distribution of up- and down-regulated genes from the *TaCESA7*-RNAi lines compared with the WT (Fielder) line. **(C)** Cluster analysis of differentially expressed genes in *TaCESA7*-RNAi lines compared with the WT (Fielder) line. Red represents high expression genes, blue represents low expression genes, clustered by RPKM values.


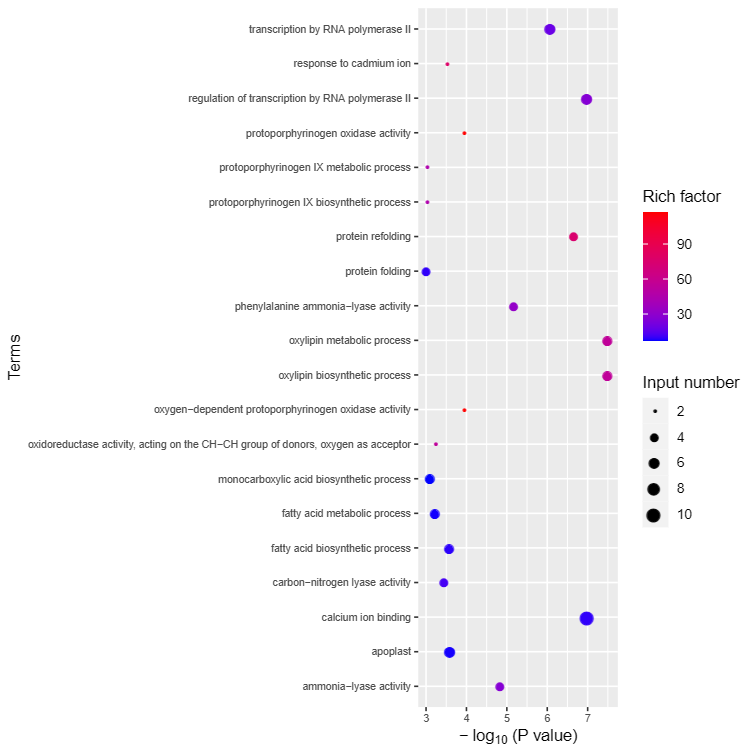


**Fig. S11** GO enrichment analysis of differentially expressed genes (DEGs) c in the *TaCESA7*-RNAi transgenic lines based on up-regulated genes.


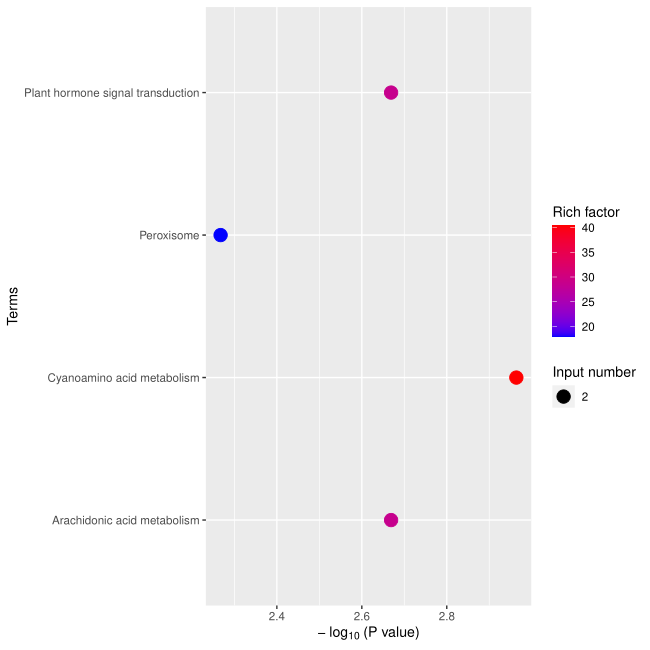


**Fig. S12** The number of differentially expressed genes (DEGs) contained in each KEGG pathway in the *TaCESA7*-RNAi transgenic lines based on down-regulated genes.

**Fig. S13** the agronomic traits and disease phenotypes inoculated with *Pst* of *TaCESA7*KO and WT plants in the field. **(A)** Wheat seeds from *TaCESA7*KO transgenic and WT plants at the mature stage. Scale bars = 2 cm. **(B-C)** Comparison of two traits in WT and *TaCESA7*KO plants growing in the field: **(B)** grain length (n = 35), and **(C)** grain width (n = 35). **(D)** The disease phenotypes of WT and *TaCESA7*KO plants growing in the field inoculated with *Pst*. Scale bars = 2 cm. **(E)** Evaluation of disease severity in WT and *TaCESA7*KO plants. Data are shown as means ± SD. Significant differences compared with WT were detected using two-tailed Student’s *t* test.
